# Alloying-Induced Structural Transition in the Promising Thermoelectric Compound CaAgSb

**DOI:** 10.1021/acs.chemmater.3c02621

**Published:** 2024-02-12

**Authors:** A. K. M. Ashiquzzaman Shawon, Weeam Guetari, Kamil Ciesielski, Rachel Orenstein, Jiaxing Qu, Sevan Chanakian, Md. Towhidur Rahman, Elif Ertekin, Eric Toberer, Alexandra Zevalkink

**Affiliations:** † Department of Chemical Engineering and Material Science, Michigan State University, East Lansing, Michigan 48824, United States; ‡ Department of Physics, Colorado School of Mines, Golden, Colorado 80401, United States; § Department of Mechanical Science and Engineering, University of Illinois at Urbana—Champaign, Urbana, Illinois 61801, United States; ∥ Department of Mechanical Engineering, Michigan State University, East Lansing, Michigan 48824, United States

## Abstract

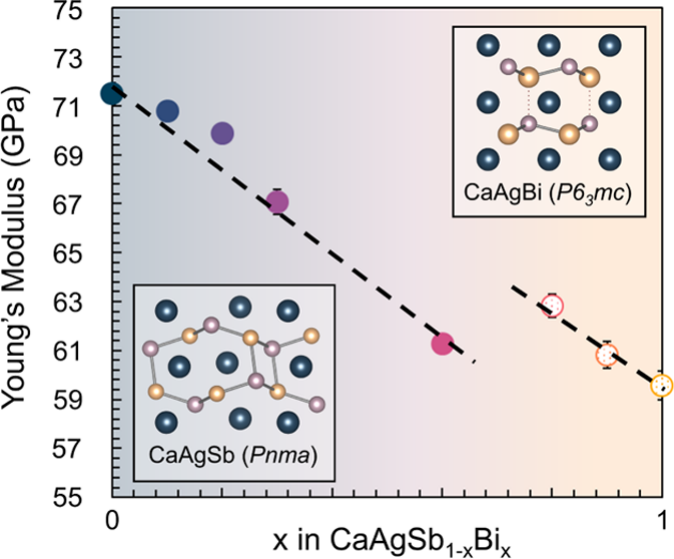

AMX Zintl compounds, crystallizing in several closely related layered structures, have recently garnered attention due to their exciting thermoelectric properties. In this study, we show that orthorhombic CaAgSb can be alloyed with hexagonal CaAgBi to achieve a solid solution with a structural transformation at *x* ∼ 0.8. This transition can be seen as a switch from three-dimensional (3D) to two-dimensional (2D) covalent bonding in which the interlayer M–X bond distances expand while the in-plane M–X distances contract. Measurements of the elastic moduli reveal that CaAgSb_1–*x*_Bi_*x*_ becomes softer with increasing Bi content, with the exception of a steplike 10% stiffening observed at the 3D-to-2D phase transition. Thermoelectric transport measurements reveal promising Hall mobility and a peak *zT* of 0.47 at 620 K for intrinsic CaAgSb, which is higher than those in previous reports for unmodified CaAgSb. However, alloying with Bi was found to increase the hole concentration beyond the optimal value, effectively lowering the *zT*. Interestingly, analysis of the thermal conductivity and electrical conductivity suggests that the Bi-rich alloys are low Lorenz-number (*L*) materials, with estimated values of *L* well below the nondegenerate limit of *L* = 1.5 × 10^–8^ W Ω K^–2^, in spite of the metallic-like transport properties. A low Lorenz number decouples lattice and electronic thermal conductivities, providing greater flexibility for enhancing thermoelectric properties.

## Introduction

Solid-state thermoelectric (TE) devices are used to convert thermal energy into electrical energy for various applications, such as deep-space probes and biomedical devices.^1,2^ The conversion efficiency in such devices directly depends on the materials parameter *zT*, known as the TE figure of merit. The dimensionless figure of merit is given by the expression , where *S* is the Seebeck coefficient, *ρ* is resistivity, *κ* represents thermal conductivity and *T* is the absolute temperature.^3^ Understanding structure–property relationships in complex materials is the key to controlling these interdependent parameters and thus to achieving high *zT*.

The term “Zintl phase” encompasses a broad family of salt-like intermetallic compounds characterized by the formal transfer of valence electrons from the cations to covalently bonded anionic substructures, effectively combining both ionic and covalent bonding in the same structure.^4−6^ Numerous Zintl compounds have been found to have excellent TE properties over the last couple decades, including but not limited to Yb_14_MnSb_11_,^7^ Mg_3_ Sb_2_,^8,9^ YbZn_2_Sb_2_,^10^ CaMg_2_Bi_2_,^11^ Ca_9_Zn_4.5_Sb_9_,^12^ KGaSb_4_,^13^ and Sr_3_GaSb_3_,^14^ etc.

*AMX* (*A* = alkali/alkaline-earth/rare-earth metal, *M* = transition metal, *X* = post-transition metalloid) Zintls are one such group that can be described by the Zintl–Klemm formalism. *AMX* compounds form several closely related crystal structures,^15^ making them an excellent space to study structure–property relationships. Of the various crystal structures, the cubic half-Heusler structure is the most extensively studied.^16,17^ The most common Zintl *AMX* structure is that of ZrBeSi, which forms a covalently bonded hexagonal *M–X* sublattice, separated by *A* cations.^18,19^ In addition to TE applications, the hexagonal *AMX* compounds like SrAgSb,^20^ EuCuSb,^21^ Eu_2_ZnSb_2_,^22^ etc. are being extensively studied as potential topological insulators^23,24^ and frustrated magnets.^25^

**Figure 1:**
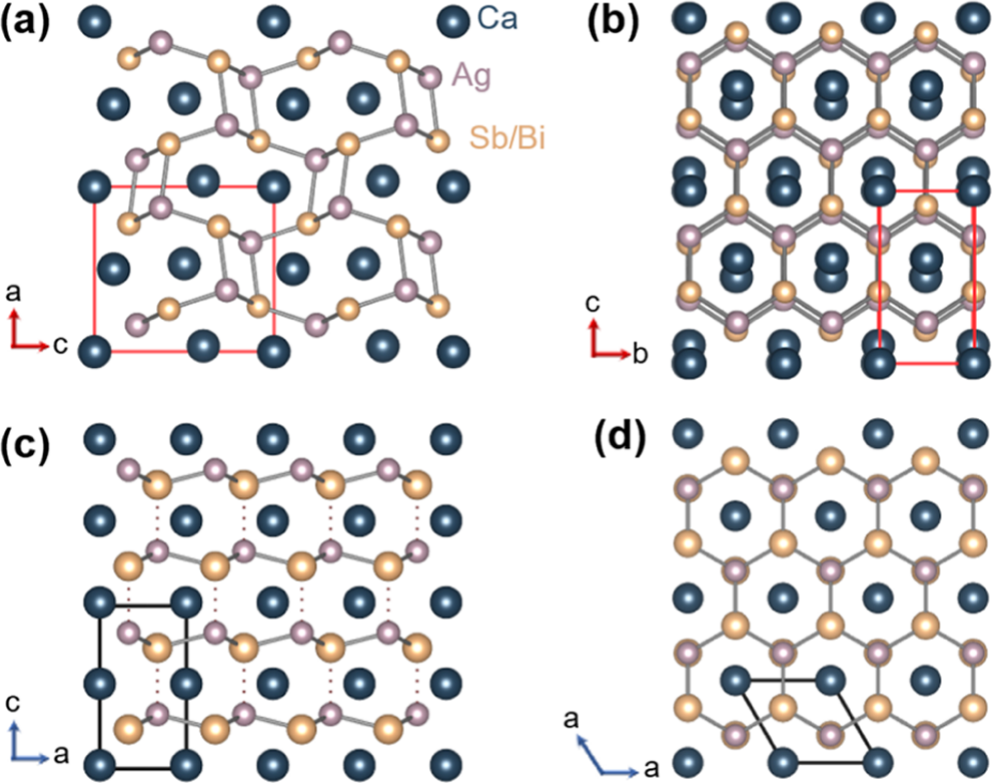
(a) Side and (b) top views of the crystal structure of orthorhombic CaAgSb, (c) side and (d) top views of the hexagonal structure of CaAgBi.

*AMX* compounds in the orthorhombic *Pnma* structure (TiNiSi-structure), shown in Figure 1a,b, have received comparatively little attention, despite over a thousand entries for the structure type in ICSD.^26^ Compared to the ZrBeSi-structure, this TiNiSi-structure type is slightly distorted, which enables covalent bonding between *M–X* layers.^27^ Recent reports of high intrinsic *zT* values in NaCdSb^28^ (*zT* = 1.3 at 673 K) and Sr_0.8_Eu_0.2_LiSb^29^ (*zT* = 1.2 at 823 K), both of which crystallize in the orthorhombic crystal structure highlight the potential of these compounds. The compound at the center of this study, CaAgSb, also forms the orthorhombic structure type. It has been shown to have a low lattice thermal conductivity, but the *zT* was largely limited by high p-type carrier concentrations.^30^ Further, CaAgSb has been shown to transform to a hexagonal *P*6_3_*mc* structure with corrugated honeycomb layers upon substitution with a small amount of La^3+^/Ce^3+^ on the Ca^2+^ site or with Zn^2+^ on the Ag^+^ site.^30,31^ The corrugated hexagonal structure is shown in Figure 1c,d. To maintain overall charge neutrality, these aliovalent substitutions also lead to the formation of vacancies at the Ag site. Such vacancies increase phonon scattering on multiple scales, leading to increased phonon scattering and lowering the lattice thermal conductivity even further.^32^ The inclusion of Zn^2+^ at the Ag^+^ site also leads to a lowering of hole concentration. Overall, this approach leads to a *zT* of ∼1 above 700 K for CaZn_0.4_Ag_0.18_Sb in the corrugated hexagonal structure.

In this work, our goal was to induce the orthorhombic-to-hexagonal structural transition in CaAgSb through isovalent alloying, instead of the aliovalent substitutions of prior studies. In this way, we gain more insight into the consequences of the structural transition by avoiding the additional complication of charged defects and vacancy formation. Hence, we alloyed CaAgSb with CaAgBi, which is known to crystallize in the corrugated hexagonal structure (*P*6_3_*mc*) and is being studied for exotic quantum applications like topological insulators.^33,34^ The complete CaAgSb_1–*x*_Bi_*x*_ (*x* = 0–1) was synthesized and characterized as a function of temperature to probe the pseudobinary phase diagram and understand the effect of the phase transition on the elastic, thermal, and electronic properties.

## Experimental Section

### Synthesis

Bulk, polycrystalline CaAgSb_1–*x*_Bi_*x*_ samples with *x* = 0.0, 0.1, 0.2, 0.3, 0.6, 0.8, 0.9, and 1.0 were synthesized through a powder metallurgy approach. Pure elements and powders were handled in an argon-filled glovebox. Stoichiometric amounts of Sb (5N plus, 99.999% purity) and/or Bi shots (Alfa-Aeser, 99.998% purity) were weighed and placed into stainless steel SPEX vials and ball milled with three 7/16″-stainless steel ball bearings for 10 min. Next, Ca dendrites (Sigma-Aldrich, 99.9% purity) and Ag shots (Sigma-Aldrich, >99.99% purity) were polished on the surface and cut into small pieces. A stoichiometric amount of metals was then weighed and added into the ball-milling vial. The resultant mixture was ball milled for 2 h using SPEX SamplePrep 8000D ball mill. The powders were scraped after the first hour of ball milling to ensure thorough mixing and then returned to the vials for the remaining hour. This ball-milling procedure was found to limit the loss of Ca and Ag (both relatively ductile) to the walls of the SPEX vials and ensure a homogeneous, stoichiometric powder. Initial synthesis attempts by simply ball-milling tiny pieces of Ca, Ag, and Sb always left the samples Ca-deficient and secondary phases of Ag_3_Sb and Sb were seen through X-ray diffraction (XRD). This is most likely due to the alkaline-earth metal getting stuck to the vial walls and refusing to come out during scraping. Therefore, ball milling the metalloids (Sb/Bi) for 10 min initially proved to be crucial. For samples *x* ≥ 0.6, 5 at. % excess Ca was added to compensate for Ca loss to the vial.

Finally, the ball-milled powders were scraped from inside the vials and poured into 10 mm diameter graphite dies with graphite foil spacers. The powders were then compacted using Spark Plasma Sintering (SPS) at 1073 K for 10 min under uniaxial pressure of 50 MPa. A heating rate of 100 K/min was used for sintering and cooling was done slowly for 30–60 min. Relative geometric densities of >94% were achieved through the sintering process. The sintered samples were found to be stable in air at room temperature.

### X-ray Diffraction

The consolidated polycrystalline samples were subsequently polished, and the phase purity was confirmed through XRD by using a Rigaku SMARTLAB diffractometer with a Cu Kα radiation source. Lattice parameters were calculated through Rietveld refinement of XRD patterns by using the Rigaku PDXL-2 software.

### Resonant Ultrasound Spectroscopy (RUS)

RUS is a nondestructive spectroscopic technique that measures the resonance frequencies of a solid with known dimensions and geometry. Instrumentation details of the RUS008 system are discussed by Migliori et al.^35^ Both room- and high-temperature spectra were collected using the open-source RUSpy software. A high-temperature stage with alumina buffer rods was used to measure the spectra under flowing argon for select samples. The heating rate was 50 K/min, and samples were held at each temperature step for 18 min. Spectra were collected for both heating and cooling cycles for every 10 K. The software RUSCal was used to extract elastic moduli by inverse numerical analysis^36^ using more than 30 peaks per spectrum. Since our samples were bulk polycrystalline with randomly oriented grains, the isotropic model was used to obtain two independent elastic constants, *c*_11_ and *c*_44_, for each sample. The elastic moduli (Young’s, shear, and bulk) and sound velocities were then calculated from *c*_11_ and *c*_44_.^37^

### Transport Properties

Thermal diffusivity (*D*) was measured by using a NETZSCH Light Flash Apparatus (LFA) 467 HyperFlash. Thermal conductivity was calculated using the equation *κ* = ϱ × *D* × *C*_v_, where ϱ is the geometric density and *C*_v_ is the constant volume heat capacity computed through the Dulong–Petit approximation. The Hall effect and electrical resistivity were measured under dynamic vacuum on a custom-built apparatus with van der Pauw geometry.^38^ The Seebeck coefficient was studied on a custom apparatus under 300 Torr N_2_ atmosphere.^39^ All electronic transport measurements were performed in heating and cooling cycles to ensure repeatability. The uncertainty of each measurement is typically found to be ∼5% which could lead to about 20% uncertainty in *zT*.

### Density Functional Theory (DFT)

The first-principles DFT calculations were performed using the plane-wave basis Vienna ab initio simulation package.^40^ The Perdew–Burke–Ernzerhof (PBE) exchange-correlation functional was used within the generalized gradient approximation.^41^ We adopted the projector-augmented wave pseudopotentials to represent core and valence electrons.^42^ The crystal structures for CaAgSb and CaAgBi were collected from ICSD.^26^ During structural relaxation, the convergence criteria for energy and forces relaxations are set as 10^–6^ eV and 10^–5^ eV Å^–1^, respectively. Electronic structures are calculated on a dense *k*-mesh with a fixed number of *k*-points. The *k*-point grid is determined according to the equation: *N*_atoms_ × *N*_kpts_ ≈ 8000, where *N*_atoms_ is the number of atoms in the primitive cell and *N*_kpts_ is the number of k-points. To address the well-documented underestimation of band gap problem in the case of semimetals and low band gap materials, the hybrid exchange-correlation function as parametrized by Heyd–Scuseria–Ernzerhof (HSE)^43^ was used to produce electron density-of-states (DOS) and carrier velocity for CaAgSb and CaAgBi. The carrier velocity is obtained using iFermi.^44^

## Results

### Phase Identification

**Figure 2:**
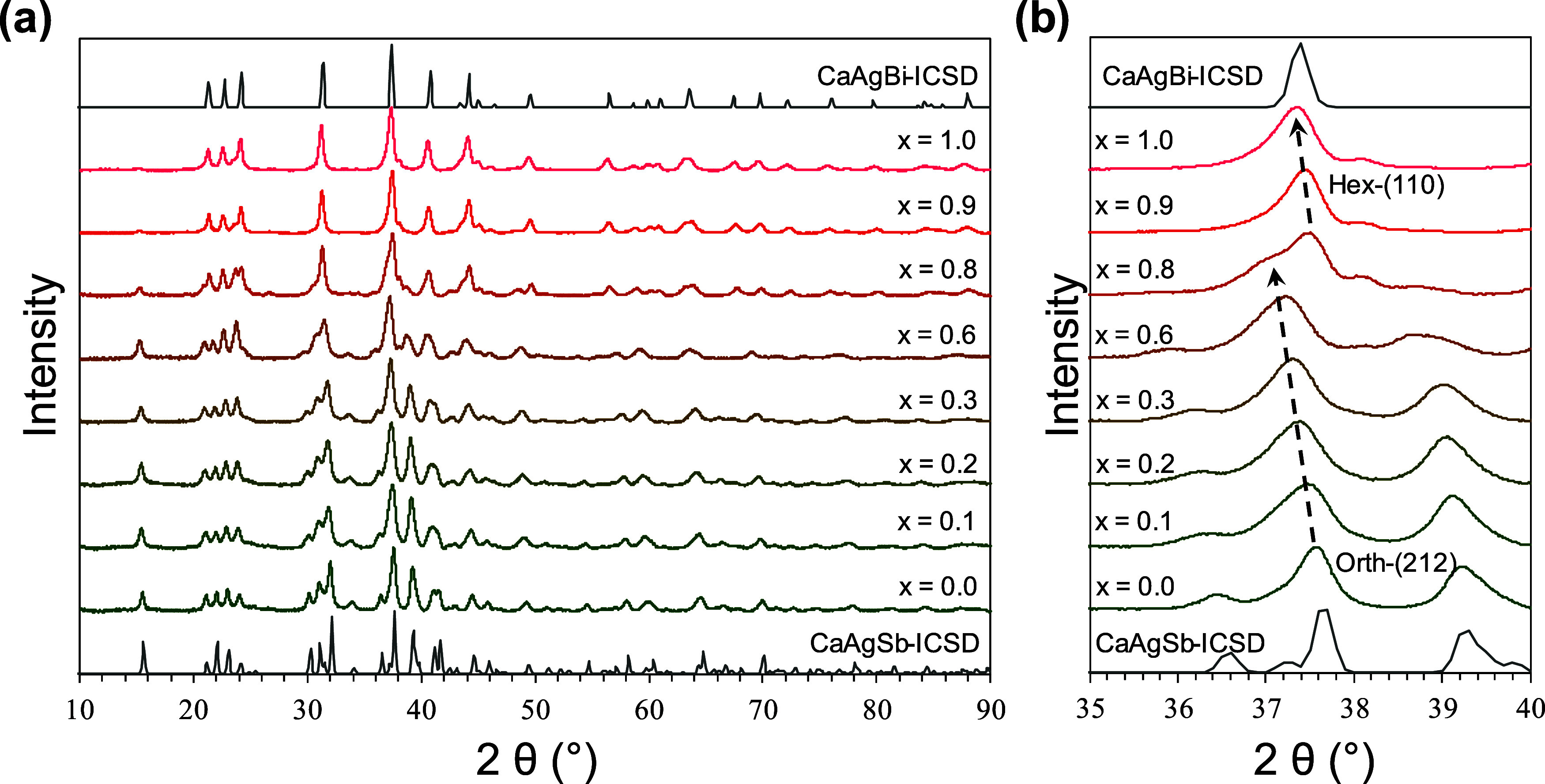
(a) X-ray diffraction patterns for CaAgSb_1–*x*_Bi_*x*_ pellets after SPS. (b) Zooming in shows the evolution of orthorhombic-(212) and hexagonal (110) peaks with increasing Bi content.

Figure 2a shows the XRD patterns for the CaAgSb_1–*x*_Bi_*x*_ as-SPS’ed pellets with *x* = 0 through *x* = 1 under ambient conditions. Up to *x* = 0.6, the samples were found to be single-phase in the orthorhombic *Pnma* structure, which was confirmed through Rietveld refinement. At *x* = 0.8, peaks for the hexagonal *P*6_3_*mc* structure began to appear alongside those of the orthorhombic structure. At *x* = 0.9 and 1.0, only the hexagonal structure was seen, in addition to some impurities. The transition from orthorhombic-to-hexagonal can be seen more easily in Figure 2b; the orth-(212) peak can be seen shifting toward lower 2θ with increasing Bi content. This is expected from the larger size of Bi (covalent radius −1.60 Å) compared to Sb (covalent radius −1.45 Å). At *x* = 0.8, both the orth-(212) and hex-(110) peaks are seen, revealing the existence of a two-phase region at the boundary. Through Rietveld refinement, the *x* = 0.8 sample was found to contain 30.47(18)% of the orthorhombic phase and 59.4(2)% of the hexagonal phase. Above *x* = 0.8, the orth-(212) peak disappears, while the hex-(110) peak continues shifting toward lower 2θ.

### Structural Transition and Elastic Properties

**Figure 3:**
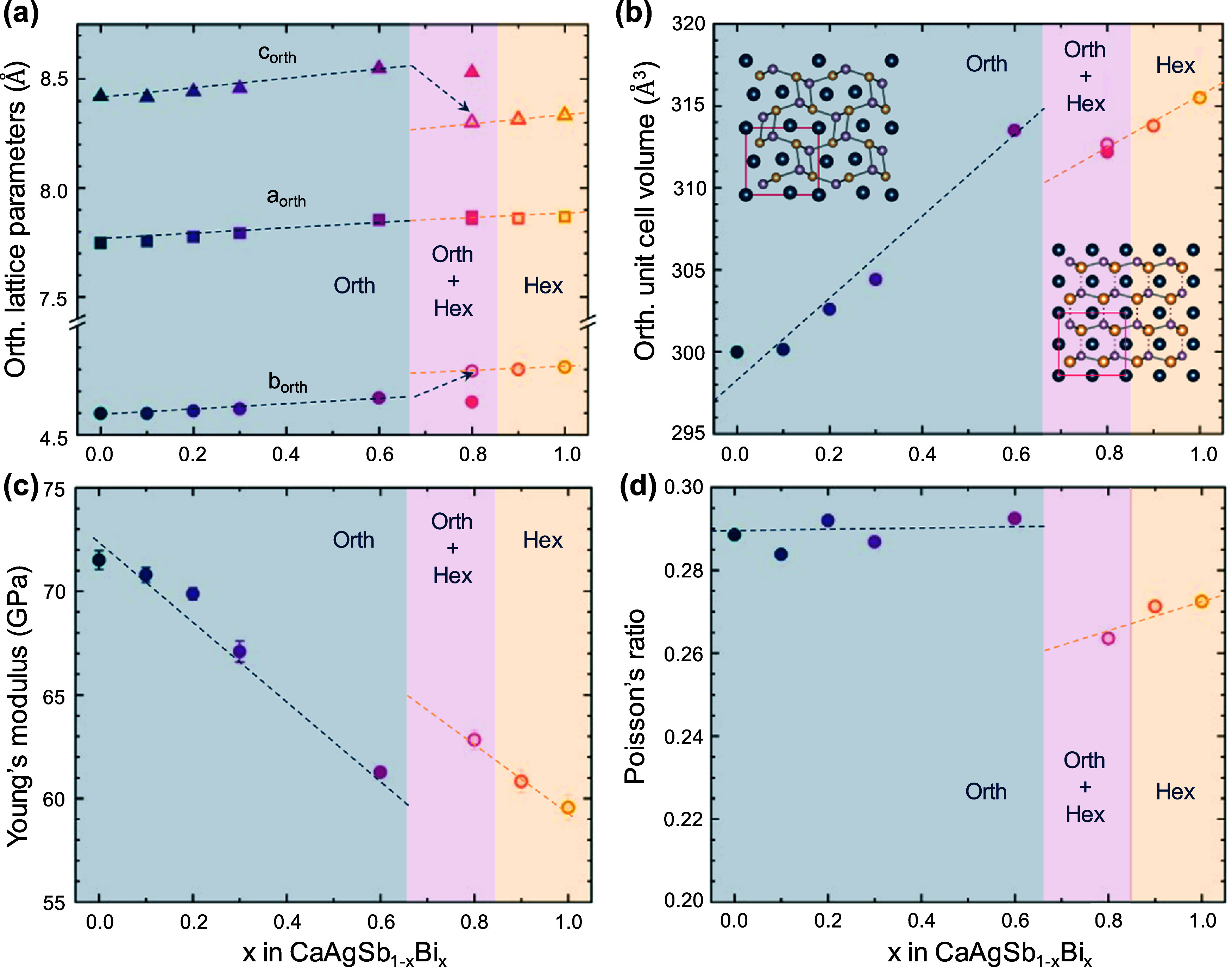
(a) Orthorhombic lattice parameters and (b) volume shown as a function of *x* calculated through Rietveld refinement. Hexagonal lattice parameters are converted to orthorhombic and are shown as open symbols. *x* = 0.8 is refined as two-phase. (c) Young’s modulus and (d) Poisson’s ratio measured using RUS at room temperature.

Figure 3a,b shows the evolution of the lattice parameters and unit cell volume of CaAgSb_1–*x*_Bi_*x*_ samples as a function of *x*. To track the changes across the phase transition, we mapped the lower-symmetry orthorhombic unit cell onto the high-symmetry hexagonal structure (see SI Figure 1 for a visual comparison). With increasing Bi content, the orthorhombic unit cell expands linearly and isotopically. At the phase transition, the lattice parameter *a*_orth_ (which is equivalent to the *c*_hex_ parameter) continues to increase linearly with no discernible discontinuity. In contrast, *c*_orth_ contracts and *b*_orth_ expand as the cell transforms to hexagonal symmetry. Overall, this transition is accompanied by a slight contraction of the unit cell volume. For *x* > 0.8, the lattice parameters continue to expand as more Bi is introduced, causing further volume expansion.

The small reduction in unit cell volume (∼1%) is found to have surprisingly large consequences; the Young’s modulus, which decreases linearly with increasing *x* due to Ag–Sb(Bi) bonds becoming larger, shows an approximately 10% increase with the crystal structure transition (Figure 3c). This suggests that the bonds, on average, are stiffer in the higher-symmetry hexagonal crystal structure than in the orthorhombic crystal structure. A similar trend is seen in the shear modulus (Figure S3). Together, the higher shear and Young’s modulus lead to a 7% increase in average sound velocity in the hexagonal phase. The Poisson’s ratio, as shown in Figure 3d, remains relatively constant within the orthorhombic regime but decreases at the phase transition, which is correlated to lowering of bulk modulus. An increase in shear-to-bulk modulus ratio is typically correlated to increased brittle behavior.^45,46^ The transverse and longitudinal sound velocities, along with the volume per atom from XRD measurements, were used to calculate the Debye temperature, glassy-limit, and diffuson limit of thermal conductivity for every composition (shown in Table S3).

The high-temperature elastic moduli were measured on select samples (*x* = 0.0, 0.1, 0.6, and 1.0), and the results for the heating cycle are shown in Figure S4. Upon heating, all of the samples show softening of bonds due to thermal expansion, which leads to a gradual, nonlinear reduction of elastic moduli. No noticeable difference is seen between the softening rate for CaAgSb vs CaAgBi. The curvature for all samples is comparable, suggesting that the Grüneisen parameter is similar across all compositions as per the Ledbetter model.^47^ Poisson’s ratio is seen to increase as a function of temperature. It is worth noting that Poisson’s ratio is typically expected to increase upon heating as samples move toward melting.^48^

### Thermoelectric and Transport Properties in CaAgSb_1–*x*_Bi_*x*_

#### Isothermal Transport Trends

**Figure 4:**
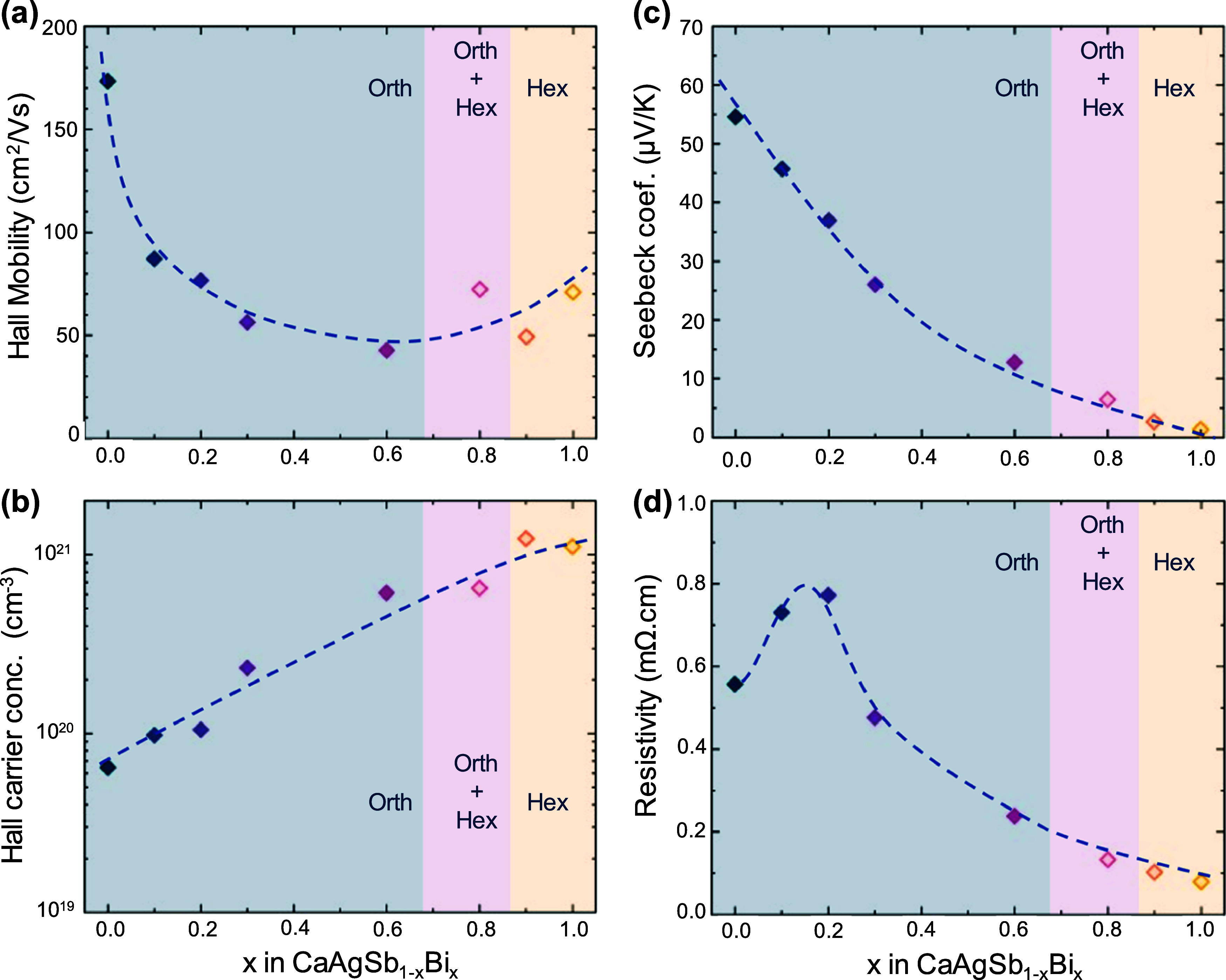
Evolution of isothermal transport properties (at 323 K) with *x* in CaAgSb_1–*x*_Bi_*x*_. (a) Hall mobility decreases due to increased scattering, while (b) carrier concentration rises. (c) Seebeck coefficient reduces due to increasing *n*. (d) Resistivity drops after an initial rise up to *x* = 0.2. Dashed curves are shown as a guide for the eye only.

The electronic and thermal transport properties measured across the CaAgSb_1–*x*_Bi_*x*_ solid solution are shown in Figure 4. All of the samples were found to be strongly p-type, despite the nominally valence-precise compositions. This suggests that an acceptor-type defect (e.g., Ca- or Ag- vacancies) might be the dominant defect type controlling carrier concentration, as seen in many other Zintls.^49,50^ The orthorhombic CaAgSb sample exhibits a promising mobility of ∼173 cm^2^/(V s) at 322 K (Figure 4a), which is comparable to high-mobility compounds such as YbMg_2_Bi_2_,^51^ EuMg_2_Bi_2_,^11^ etc. Substitution of 10 atom % Bi at the Sb site (*x* = 0.1) decreases the mobility by half, likely arising from substitutional-defect scattering. Upon further Bi substitution, the mobility decreases even further, before increasing slightly in the hexagonal structure. The Hall carrier concentration, shown in Figure 4b, increases exponentially with increasing Bi substitution from 6 × 10^19^ cm^–3^ in CaAgSb to ∼10^21^ cm^–3^ in CaAgBi. The increased carrier concentration suggests a strong reduction in defect formation energy or the introduction of a new dominant defect type due to the introduction of Bi.

The Seebeck coefficient, shown in Figure 4c, also decreases with an increasing Bi content, driven by the increasing carrier concentration. Resistivity, however, increases from *x* = 0 to 0.2 and then decreases upon further Sb substitution (Figure 4d). The initial increase in resistivity comes from the large reduction in mobility in comparison to the slight increase in carrier concentration for *x* ≤ 0.2. As the mobility levels off, the rising carrier concentration dominates, leading to decreasing resistivity. The decreasing Seebeck coefficient and resistivity also point toward a narrowing (or closing) band gap with increasing Bi substitution.

#### Temperature-Dependent Transport

**Figure 5:**
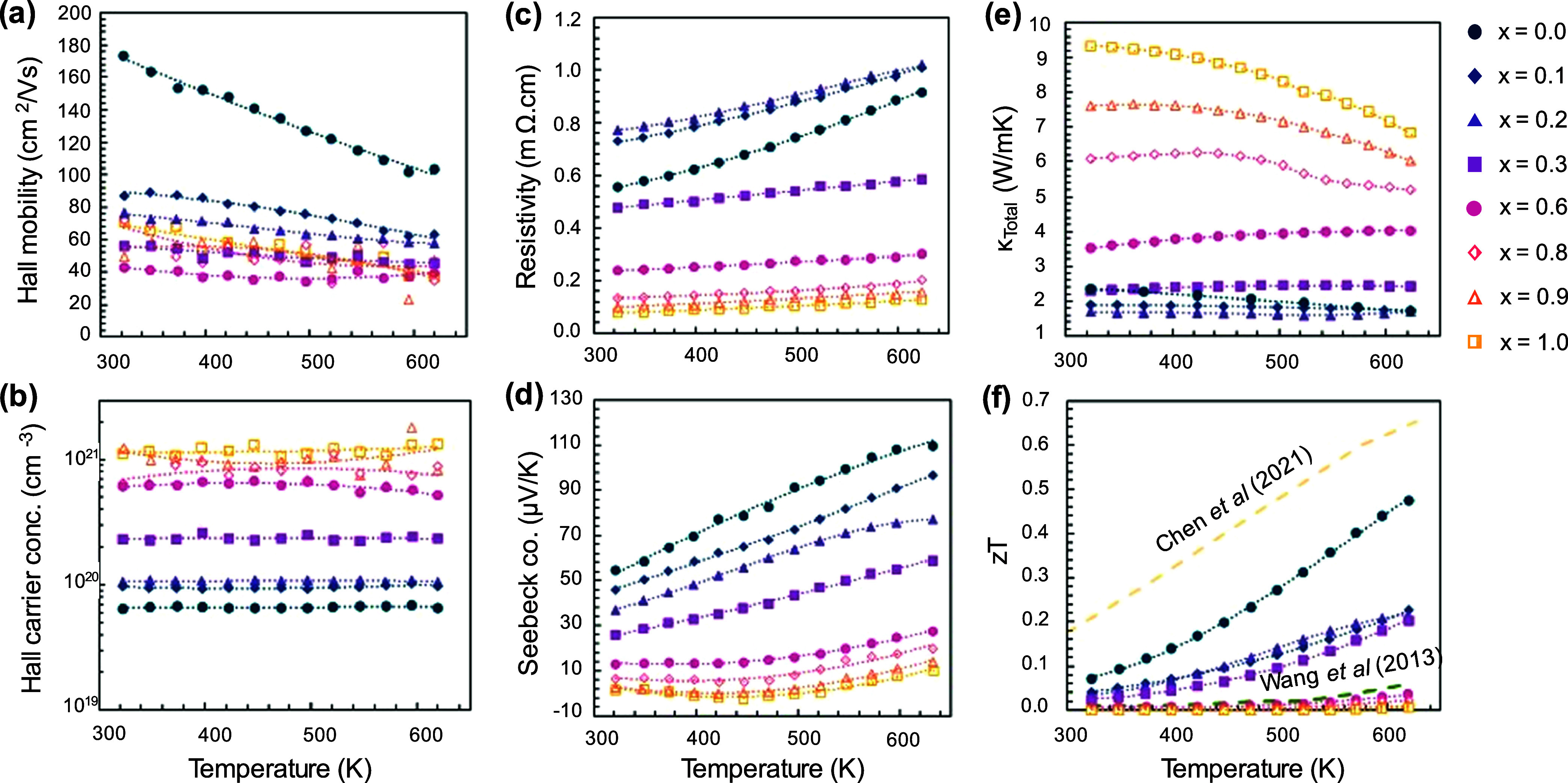
Temperature-dependent (a) mobility, (b) carrier concentration, (c) resistivity, (d) Seebeck coefficient, (e) total thermal conductivity, and (f) *zT* values shown across all compositions. For comparison, Wang et al. (CaAgSb) and Chen et al. (CaAg_0.2_Zn_0.4_Sb) *zT* results have also been shown. The results are consistent with what can be expected for the CaAgSb_1–*x*_Bi_*x*_ series. The data shown here were collected during heating. For both heating and cooling data, see Figure S5.

As a function of increasing temperature, the mobility in the end member *x* = 0 decreases sharply (Figure 5a); the quasi-linear decline can be correlated with acoustic phonon scattering in degenerate regime.^52^ Upon substitution, the mobility trend begins to flatten, which is consistent with alloy scattering. The carrier concentration remains mostly independent of the temperature across the series (Figure 5b). The resistivity (Figure 5c) and Seebeck coefficients (Figure 5d) increase with temperature, typical of degenerate semiconductors. The temperature-dependent thermal conductivity, *κ*, is shown in Figure 5e. With increasing Bi content, *κ* decreases from 2.4 W/mK at 323 K to 1.6 W/mK for *x* = 0.2, likely caused by alloy scattering-induced reduction of both lattice (*κ*_L_) and electronic (*κ*_E_) contributions. For *x* > 0.2, *κ* increases significantly as a function of *x*, consistent with the decrease in resistivity. The trends in *κ*_L_ and *κ*_E_ are discussed in depth in the Lattice Thermal Conductivity and Lorenz Number Approximations Section.

#### Single Parabolic Band Model

**Figure 6:**
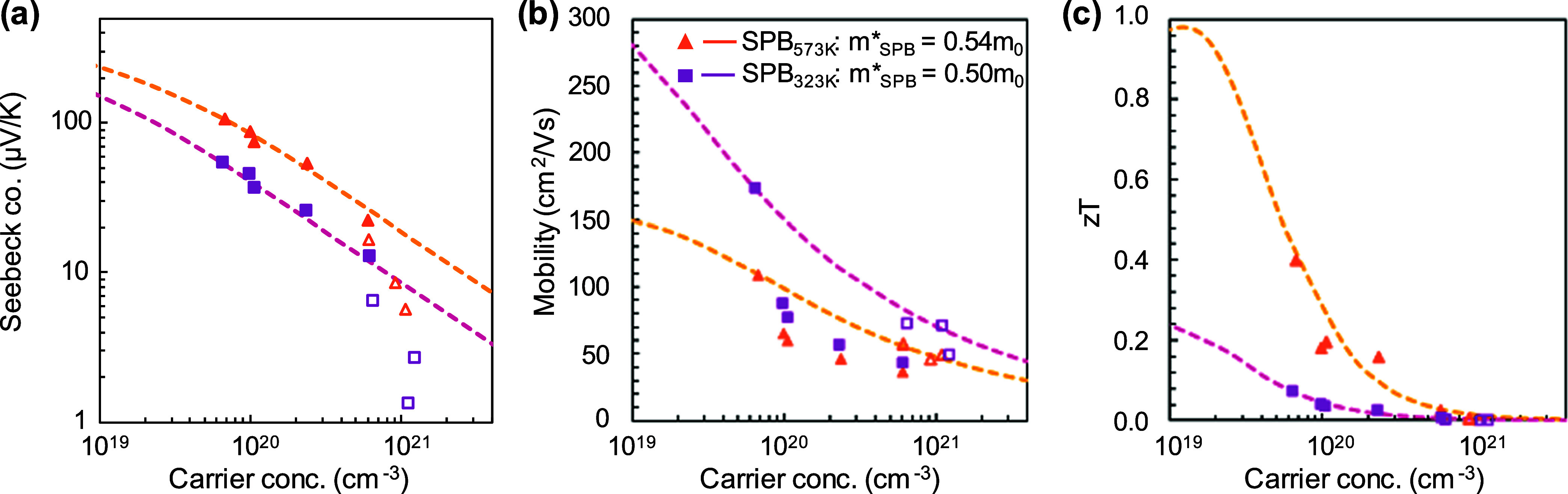
Pisarenko plot for (a) Seebeck coefficient at 323 and 573 K suggest that samples in the orthorhombic crystal structure have effective masses of 0.50 and 0.54 m_0_ respectively. (b) Pisarenko plot of Hall mobility shows that the mobility decreases with increasing carrier concentration at both temperatures, suggesting that, for the end members, *x* = 0 and 1, acoustic deformation potential scattering might be the dominant form of scattering at both 323 and 573 K. The alloyed samples have mobility that dips well below the SPB model, indicating additional scattering due to alloy scattering. (c) Carrier concentration-dependent *zT* plot suggests that a *zT* of 1 at 573 K can be achieved if the carrier concentration of 10^19^ cm^–3^ can be reached, assuming that acoustic deformation potential scattering is the dominant form of scattering.

Figure 6a shows the Seebeck coefficient as a function of the hole concentration. The single parabolic band (SPB) model with an assumption of acoustic phonon scattering^53−55^ was used to calculate the effective mass, m*_SPB_. For the CaAgSb end member, m*_SPB_ is 0.50 and 0.54 m_0_ at 323 and 573 K respectively, which is slightly lower than that found in many AM_2_X_2_ Zintl compounds.^56,57^ For all CaAgSb_1–*x*_Bi_*x*_ samples in the orthorhombic structure, the experimental data suggest no change to m*_SPB_ as a function of *x*, as highlighted by the dashed line in panel Figure 6a. However, for the samples with the hexagonal structure (shown as hollow markers), the Seebeck coefficients are well below the SPB model prediction. The deviation might be either due to a lowering of effective mass due to crystal transition or due to multiband transport characteristic of the more-or-less metallic Bi-rich samples. The mobility as a function of carrier concentration is shown in Figure 6b. The reduction of mobility with increasing carrier concentration is consistent with the SPB assumptions of acoustic deformation potential scattering for the end members *x* = 0.0 and 1.0. Deviation from the SPB model is seen for both temperatures for alloyed samples, which is likely due to alloy scattering from substitutional defects.

#### Figure of Merit

Finally, Figure 6c shows the evolution of *zT* with carrier concentration (*n*) at 323 and 573 K. The SPB model predicts that a *zT* of 1 can be reached at 573 K at *n* ∼ 10^19^ cm^–3^. However, all of the samples in the present study have carrier concentrations well above the optimal value. CaAgSb has the lowest hole concentration, and increasing the Bi content increases *n*. Prior literature suggests that *V*_Ag_ is the dominant form of defect in CaAgSb, which might be driving the high carrier concentration across this series of samples.^30,58^ Tuning the Ag content or introducing compensating n-type dopants can potentially be a possible way to optimize the thermoelectric properties of orthorhombic CaAgSb.

In the absence of an optimized carrier concentration, however, we conclude that Bi substitution is not beneficial for the figure of merit. The highest figure of merit in the present series is found to be 0.47 at 620 K in unmodified CaAgSb (Figure 5f). We note, however, that this *zT* value is substantially higher than the only previous report of *zT* in unmodified CaAgSb.^30^ In comparison to the prior work, which looked at flux-grown CaAgSb, the samples synthesized in this work appear to have a lower hole carrier concentration based on their higher Seebeck coefficient, higher resistivity, and lower thermal conductivity (see Figure S5 for a comparison). To date, the highest *zT* reported for CaAgSb-based materials is that of vacancy-rich CaZn_0.4_Ag_0.18_Sb,^59^ which actually forms the hexagonal structure. Its higher *zT* can be attributed to increased phonon scattering and reduced carrier concentration.^32^

#### Density Functional Theory

**Figure 7:**
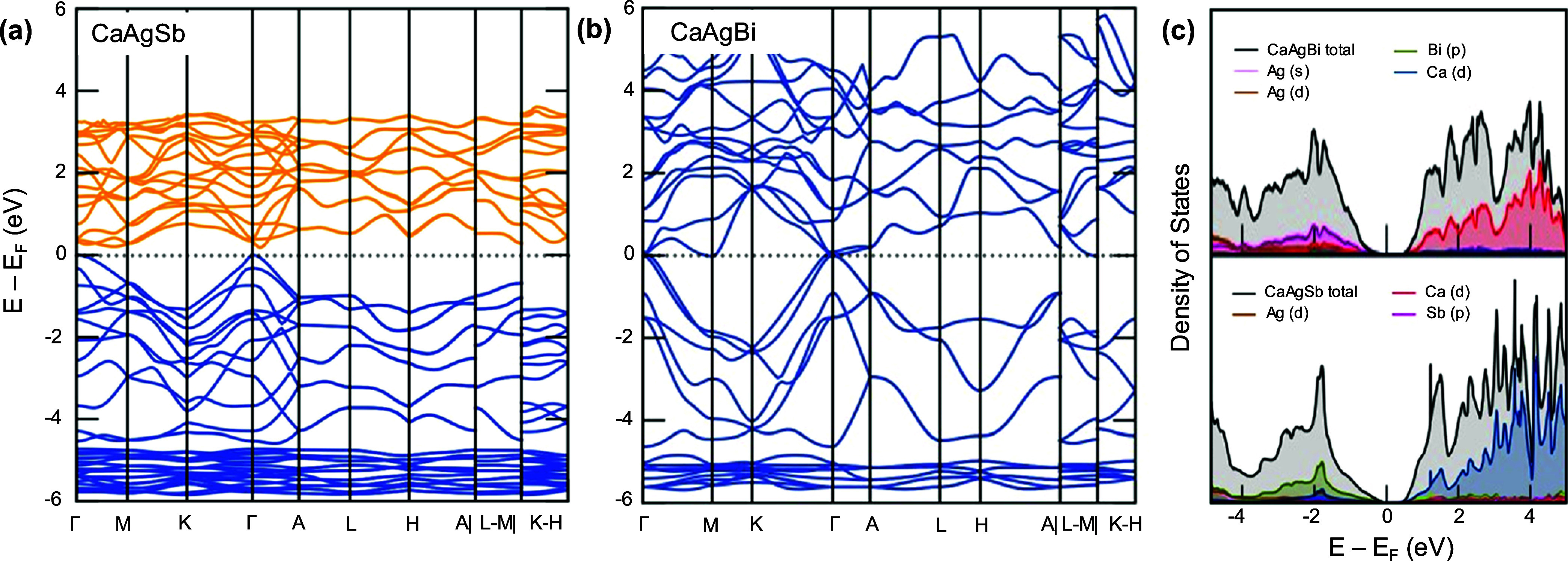
Band structure along all *k*-points for (a) CaAgSb and (b) CaAgBi using PBE functionals. (c) Partial and total DOS values for the two compounds calculated using HSE functionals reveal narrow band gaps.

In order to better understand the transport properties, the electronic band structures and density of states were calculated for the two end members, as shown in Figure 7. The band structures in Figure 7a,b show a small indirect band gap (∼0.1 eV) in CaAgSb, and no band gap in CaAgBi. The density of states in Figure 7c use HSE corrections, reveal band gaps of 0.40 and 0.16 eV for CaAgSb and CaAgBi, respectively. In CaAgSb, the valence band at Γ dominates the transport properties, with another flatter band lying ∼0.5 eV lower. Band structure calculations reveal that another likely route to improve TE properties of CaAgSb would be band convergence at the Γ point, which could lead to increased Seebeck coefficients. The conduction band minimum is between Γ and A, suggesting an indirect band gap. In the Bi analogue, the two nearby valence bands seem to have converged at the Γ point. However, due to a small band gap and possible mixed conduction, we do not see an increased Seebeck coefficient as one would expect from band convergence. Partial DOS (pDOS) reveals that the conduction bands are dominated by Ca, while the valence band is dominated by the nonmetal atom (Sb/Bi).

### Lattice Thermal Conductivity and Lorenz Number Approximations

#### SPB-Based Lorenz Number

Heat transport in semiconductors primarily occurs through electronic carriers and lattice contributions. The electron (or hole) contribution to thermal conductivity *κ*_E_ is typically estimated using the Wiedemann–Franz Law, which states that *κ*_E_ is directly proportional to electrical conductivity (*σ* = 1/*ρ*) related by a proportionality constant called the Lorenz number. The lattice contributions are found by subtracting *κ*_E_ from the total *κ*. The Lorenz number, which is a key factor in *κ*_E_, depends on the position of the Fermi energy, *E*_F_, and the details of the density of states and carrier relaxation time near *E*_F_.^60^ Within the SPB model, the Lorenz number is often approximated as *L*_SPB_ = 1.5 + exp(−|*S|*/116), where *S* denotes the Seebeck coefficient.^61^ In essence, this approximation limits the Lorenz number between the values of 1.5 × 10^–8^ W Ω K^–2^ (for nondegenerate insulators) and 2.5 × 10^–8^ W Ω K^–2^ (approximate limit for metals or degenerate semiconductors).^62,63^ While this approximation works well for many material systems, in the context of compounds with complex, nonparabolic band structures or small band gaps, the *L*_SPB_ method can significantly diverge from the true *L* value.^63^

**Figure 8:**
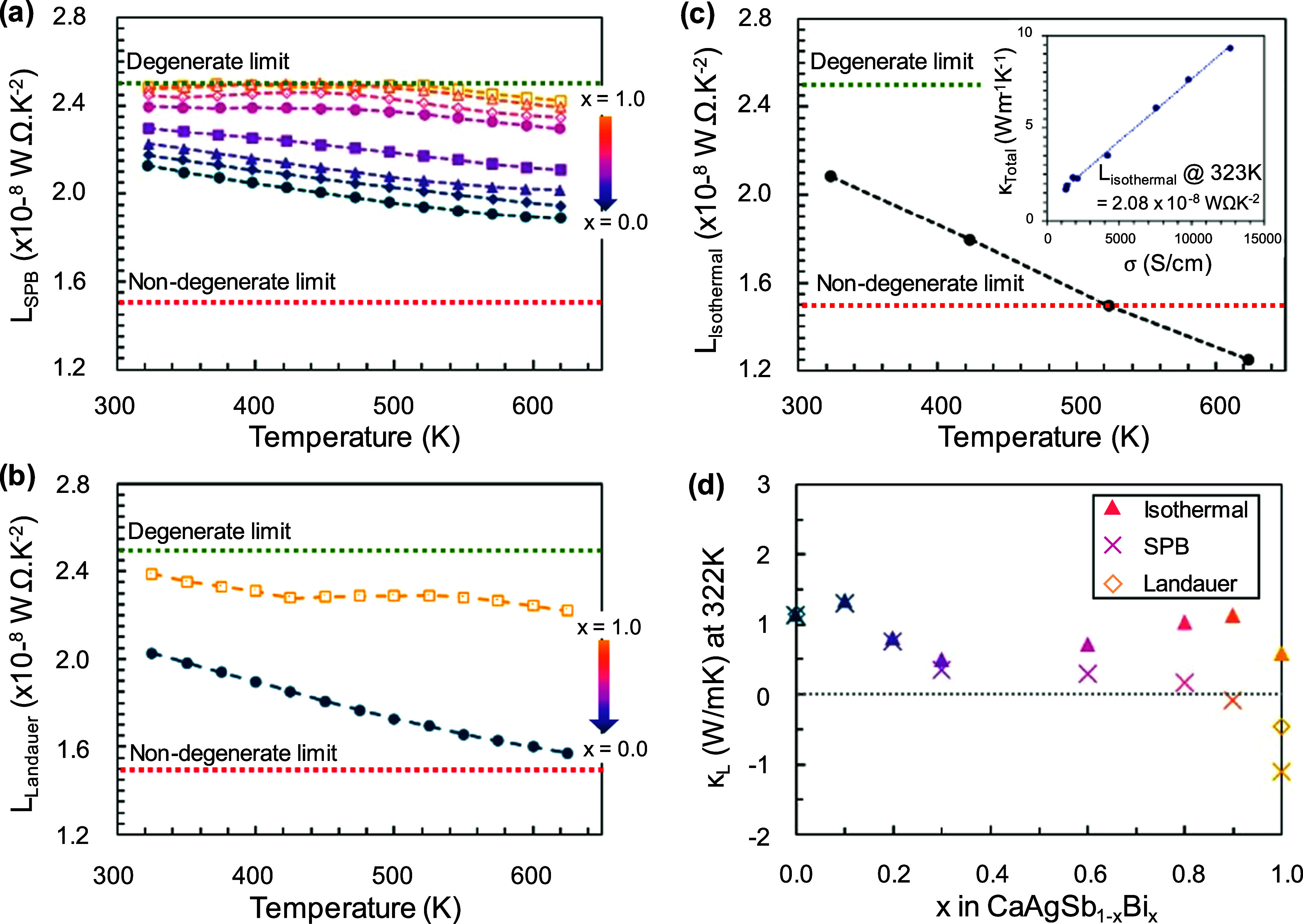
Temperature-dependent Lorenz number calculated using (a) SPB model, (b) modified Landauer model, and (c) Isothermal model. (d) The Lorenz number using the three approaches was used to calculate *κ*_L_ across all compositions at 323 K.

We began by calculating *κ*_E_ using *κ*_E_ = *σ* × *T* × *L*_SPB_, which, for pure CaAgSb, yielded reasonable values for *κ*_E_ and *κ*_L_, consistent with prior literature reports.^30^ As the samples become more metallic (the band gap is reduced and the carrier concentration increases) with increasing Bi content, the *L*_SPB_ derived from the Seebeck coefficient becomes higher, reaching the degenerate limit, as shown in Figure 8a. This yields unrealistically high values of *κ*_E_, which exceeds *κ*_total_ (Figure S8). Consequently, for all samples with *x* ≥ 0.2, the so-derived *κ*_L_ values are predicted to be negative at elevated temperatures. In the most extreme case of *x* = 1.0, *κ*_L_ is found to reach a minimum of −5 W m^–1^ K^–1^ at 620 K (Figure S8). These results are clearly unphysical, implying that the SPB model significantly overestimates *L* in the Bi-rich alloys.

#### Landauer-Based Lorenz Number

In search of reasonable values for lattice thermal conductivity, we next employed a Landauer formalism with input from first principles.^64^ Details of this approach can be found in SI Section 9. The energy-dependent electron DOS and velocities were calculated from first-principles DFT using HSE functionals for CaAgSb (*Pnma*) and CaAgBi (*P*6_3_*mc*). The energy-dependent carrier relaxation time, τ*(E)* was presumed to exhibit an inverse correlation with the magnitude of the DOS. There are two unknown temperature-dependent variables: Fermi level (*E*_F_) and the carrier relaxation time scaling factor (*τ*_0_). These were obtained by solving the Landauer equations for the Seebeck coefficient and electrical conductivity using experimental *S* and *σ* as input. Finally, these variables were used to calculate the Lorenz number using the Wiedemann–Franz law.^65^ The so-derived Lorenz numbers, denoted as *L*_Landauer_, are shown in Figure 8b as functions of temperature for both CaAgSb and CaAgBi. For both, the *L*_Landauer_ predicts values slightly lower than that predicted by using the SPB model (*L*_SPB_). This leads to reasonable lattice thermal conductivity in CaAgSb (see Supporting Information), but on the Bi side, *κ*_E_ is still > *κ*_total_. These analyses leave some open questions on the CaAgBi side and our theoretical models to understand Lorenz number in narrow band gap semiconductors with complex electronic structures.

#### Isothermal Lorenz Number Model

As a third approach to determine the Lorenz number, we applied the Wiedemann–Franz law (κ_total_ = κ_L_ + σ × *T* × *L*) to directly fit *κ*_total_ as a function of *σ*. At a fixed temperature (isothermal condition), the Wiedemann–Franz law describes a linear *κ*_total_ vs *σ* relationship, with a *y*-intercept of *κ*_L_ and a slope equal to *L*_isothermal_ × *T*. This approach would typically be applied for a series of materials that have the same or similar value of *L* but differing magnitudes of *κ*_E_. Early models for the Lorenz number were designed based on this isothermal approach,^66^ and this direct approach has been used in several recent probes of the experimental Lorenz number of bismuth antimony telluride.^67^ A similar approach was also introduced by Lukas et al. for doped bismuth telluride and bismuth–antimony alloys.^68^

Here, we find that, at room temperature, a plot of *κ*_total_ vs *σ* for the samples in the CaAgSb_1–*x*_Bi_*x*_ series yields a linear trend (Figure 8c inset), suggesting that the *L* does not vary strongly at room temperature as a function of either Bi content or crystal structure. The Lorenz number obtained from the slope at room temperature is *L*_323 K_ = 2.08 × 10^–8^ W Ω K^–2^. Repeating this process at various fixed temperatures yields the temperature-dependent (but composition independent) *L*_isothermal_ values shown in Figure 8c. The fits of *κ*_total_ vs *σ* are shown for each temperature in Figure S11. The isothermal linear fits are in excellent agreement with experimental data despite the crystal transition. Using this approach, a linear decrease in *L*_isothermal_ is seen as a function of temperature, and *L*_isothermal_ reaches a value of 1.25 × 10^–8^ W Ω K^–2^ at 623 K. This is much lower than the nondegenerate semiconductor limit set by theoretical models.

#### Comparison of *κ*_L_ with Three Models for *L*

Figure 8d shows a comparison of *κ*_L_ calculated using the three models for *L* across the CaAgSb_1–*x*_Bi_*x*_ series at 323 K. Only the isothermal approach yields positive values of *κ*_L_ across the entire CaAgSb_1–*x*_Bi_*x*_ series at all temperatures (See Figure S11 for temperature-dependent trends). Using *L*_isothermal_, low lattice thermal conductivity is observed across the series, as evident by the *x* = 0.3 sample reaching the diffuson limit for a minimum *κ*_L_. Note that an unexpected slight increase in *κ*_L_ is seen with 0.1 addition of Bi at the Sb site, regardless of the Lorenz number model used.

The agreement among the three models for *L* is a strong function of composition. Figure 7d shows that for *x* = 0 to 0.3, all three models show reasonable agreement for *κ*_L_. For the Bi-rich compositions, we begin to see significant divergence between the SPB and Landauer models on the one side and the isothermal model on the other. There are two factors that lead to the stronger divergence in estimated *κ*_L_, (1) with increasing *x*, the predicted values of *L*_SPB_ and *L*_Landauer_ become larger, while *L*_isothermal_ is constant. (2) with increasing *x*, electrical conductivity increase, which means that the differences in *L* have a greater consequence for *κ*_E_ and *κ*_L_.

Further, the experimentally determined Lorenz number was used to calculate *κ*_L_ for all values of *x* and *T* (see Figure S12). The results show positive values of the lattice thermal conductivities across the CaAgSb_1–*x*_Bi_*x*_ series at all temperatures. Extremely low lattice thermal conductivity is observed across the series, as evident by the *x* = 0.3 sample reaching the diffuson limit for minimum *κ*_L_. The temperature-dependent trends look somewhat unorthodox, especially on the Bi-rich side. This may be due to a very small band gap coupled with multiband-multicarrier transport in these samples. The underlying reasons for such low Lorenz numbers on the CaAgBi-side warrant further investigation.

Observations of negative lattice thermal conductivity, especially in low *κ*_L_ materials with high electrical conductivity, are widespread in literature,^69,70^ and this study highlights the importance of rigorous Lorenz number analysis. At the moment, most studies rely on an SPB-based Lorenz number to experimentally distinguish between lattice (phonon) and electronic contributions to the thermal conductivity. However, prior experimental and theoretical studies have also revealed Lorenz numbers well below the nondegenerate theoretical limit.^55,63,71^ Therefore, more rigorous theoretical models need to be developed in order to advance our understanding of the underlying structure–property relationship and to better design thermoelectric materials.

## Conclusions

The transition from orthorhombic (*Pnma*) to hexagonal (*P*6_3_*mc*) crystal structure was successfully achieved through alloying within the CaAgSb_1–*x*_Bi_*x*_ series, with XRD confirming the evolution. We found that while adding Bi softens the elastic constants, there is an unexpected jump in stiffness at the phase transition to the hexagonal structure. The orthorhombic CaAgSb sample exhibited a promising intrinsic thermoelectric figure of merit, *zT* = 0.47. By fitting experimental data to a single parabolic band model (SPB), we determined an effective mass of 0.50 m_0_ at 323 K for CaAgSb, which appeared to be insensitive to Bi content as long as the orthorhombic phase was maintained. Predictions from the SPB model suggested a potential *zT* value of 1 at 573 K in the orthorhombic structure with reduced carrier concentration. Assessing the lattice thermal conductivity (*κ*_L_) accurately was challenging in this system. Negative *κ*_L_ values were obtained via the SPB model and Landauer formalism approximation in the Bi-rich portion of the series. However, an isothermal Lorenz number model provided physically reasonable *κ*_L_ values in the range of 0.5–1.3 W mK^–1^ at 323 K. In general, there remains a pressing need for rigorous theoretical models for the Lorenz number in complex materials. Materials that combine extremely low thermal conductivity and high electronic conductivity, like CaAgBi, provide an ideal platform for comparing and developing improved Lorenz number models.

## Supporting Information Available

Rietveld analysis results, temperature, and composition-dependent properties; multiple models for Lorenz number and analysis of anisotropy in transport properties (PDF)
