# Supplementary material for: Alloying-Induced Structural Transition in the Promising Thermoelectric Compound CaAgSb
Source: Chem Mater. 2024 Feb 12;36(4):1908–18. doi: 10.1021/acs.chemmater.3c02621 (PMC10961731; doi:10.1021/acs.chemmater.3c02621)
Supplement: Supplementary file 1 — cm3c02621_si_001.pdf [file cm3c02621_si_001.pdf]

Supporting Information - Alloying-induced structural transition in the promising thermoelectric compound CaAgSb

A. K. M. Ashiquzzaman Shawon[1], Weeam Guetari[1], Kamil Ciesielski[2], Rachel Orenstein[2], Jiaxing Qu[3], Sevan Chanakian[1], Md. Towhidur Rahman[4], Elif Ertekin[3], Eric Toberer[2], Alexandra Zevalkink[1]*

[1] Department of Chemical Engineering and Material Science, Michigan State University, East Lansing, Michigan 48824, United States

[2] Department of Physics, Colorado School of Mines, Golden, Colorado 80401, United States

[3] Department of Mechanical Science and Engineering, University of Illinois at Urbana-Champaign, Urbana, Illinois 61801, United States

[4] Department of Mechanical Engineering, Michigan State University, East Lansing, Michigan 48824, United States

Table of Contents

Section #	Section name	Page no.
**SI 1**	Lattice parameter conversion from hexagonal to orthorhombic	2
**SI 2**	Rietveld refinement and lattice parameters	3
**SI 3**	Elastic constants vs composition and temperature	6
**SI 4**	Debye temperature and glassy limit	8
**SI 5**	Comparison of thermoelectric transport properties of CaAgSb	9
**SI 6**	Reversibility of transport properties	10
**SI 7**	Spin-orbit coupling effects on band structure	11
**SI 8**	Electronic and lattice thermal conductivity estimated using the SPB model	12
**SI 9**	Landauer formalism to get Lorenz number	13
**SI 10**	Isothermal Lorenz number	16
**SI 11**	Electronic transport along different directions	18
	References	20

SI 1: Lattice parameter conversion from hexagonal to orthorhombic

To uniformly compare the changes in unit cell size as a function of composition, the lower symmetry orthorhombic lattice parameters are used. Figure S1 shows the hexagonal *P6₃mc* crystal structure, where the blue lines represent the hexagonal *P6₃mc* unit cell, while the red lines represent orthorhombic *Pnma* unit cell. The arrows represent respective crystallographic axes. The equations on the right can be applied to convert the hexagonal lattice parameters into the orthorhombic lattice parameters.

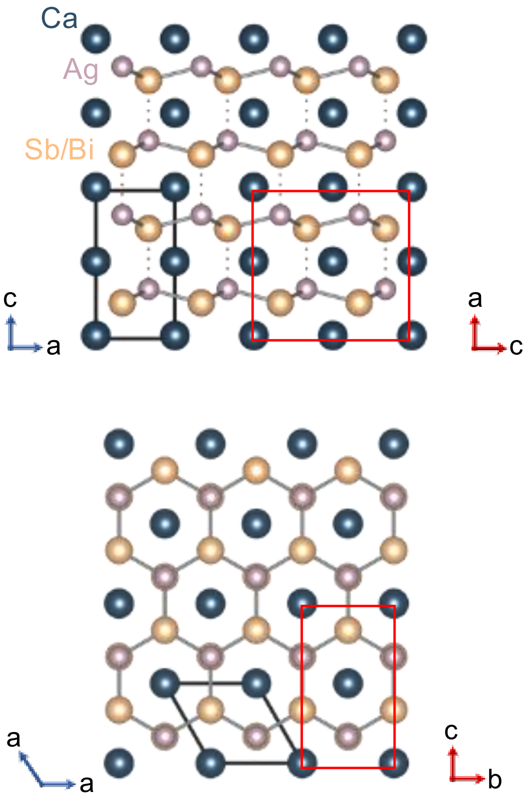

$$a_{orth} = c_{hex}$$
$$b_{orth} = a_{hex}$$
$$c_{orth} = 2 \, . \, a_{hex} \, . \, \cos(30^{o})$$

Figure S1: The orthorhombic unit cell (red) mapped on the hexagonal crystal structure. The hexagonal lattice parameters can be converted to orthorhombic lattice parameters using the equations shown above. Note that the volume of the orthorhombic cell, $V_{orth} = 2 \times V_{hex}$

SI 2: Rietveld refinement and lattice parameters

Rietveld refinements were performed on as-SPS'ed pellets to calculate lattice parameters. The results are summarized for samples in the orthorhombic crystal structure (*Pnma*) and for samples with the hexagonal crystal structure ($P6_3mc$) in SI Tables 1 and 2 respectively. SI Figure 2 shows the refinement fits for each sample.

Table S1: Rietveld refinement results for samples in the orthorhombic (*Pnma*) crystal structure:

**Sample**	***x* = 0.0**	***x* = 0.1**	***x* = 0.2**	***x* = 0.3**	***x* = 0.6**
Nominal composition	CaAgSb	$CaAgSb_{0.9}Bi_{0.1}$	$CaAgSb_{0.8}Bi_{0.2}$	$CaAgSb_{0.7}Bi_{0.3}$	$Ca_{1.05}AgSb_{0.4}Bi_{0.6}$
Rwp (%)	2.93	2.56	2.47	2.70	3.26
Phases	*Pnma*	*Pnma*	*Pnma*	*Pnma*	*Pnma*
Pnma<br>Lattice parameters (Å)	a: 7.748238<br>b: 4.597716<br>c: 8.420799	a: 7.7558(10)<br>b: 4.5973(7)<br>c: 8.4175(8)	a: 7.7762(10)<br>b: 4.6091(7)<br>c: 8.4426(8)	a: 7.7928(10)<br>b: 4.6189(7)<br>c: 8.4573(8)	a: 7.854(3)<br>b: 4.6689(15)<br>c: 8.4594(12)

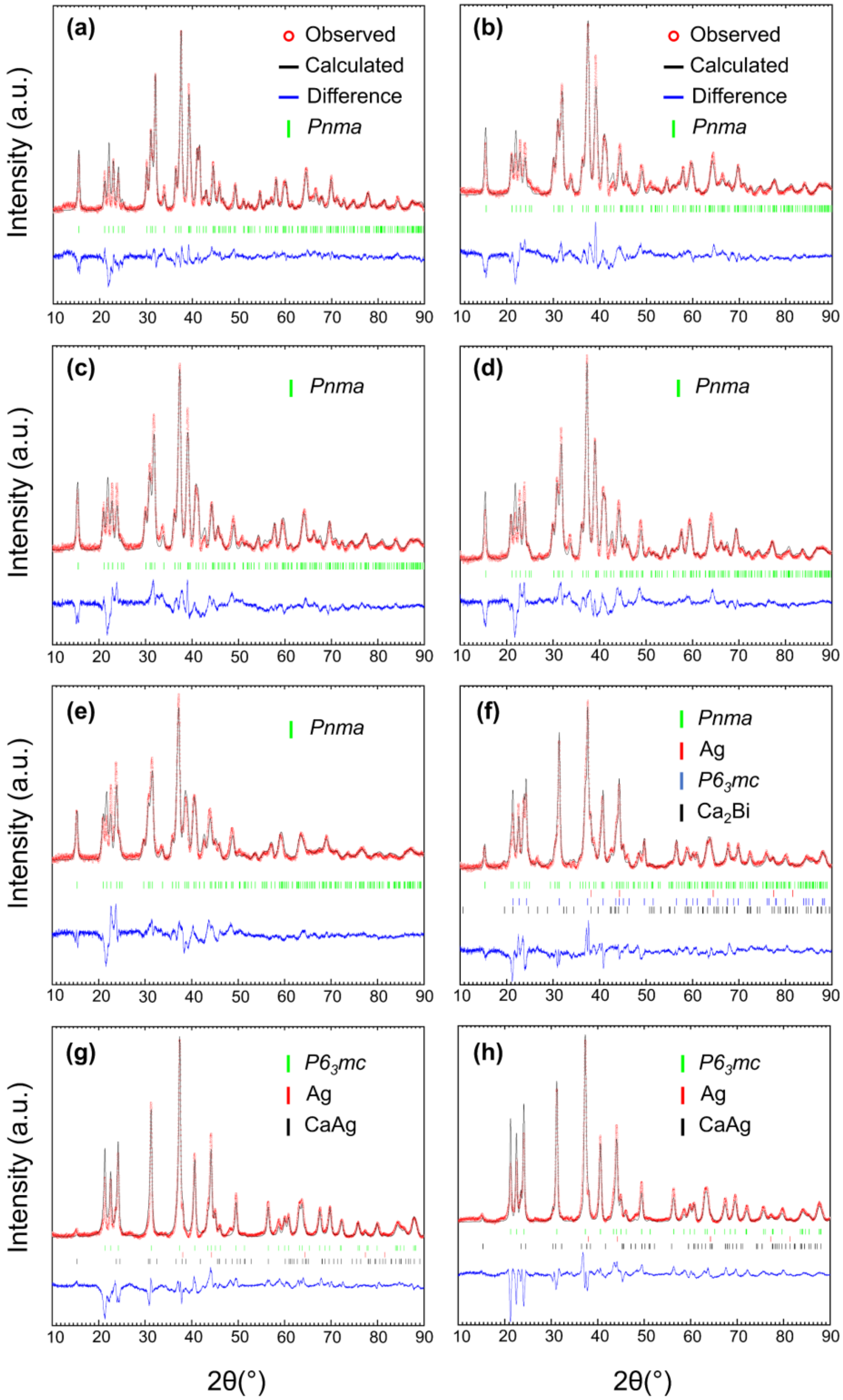

Figure S2: Rietveld refinement fits for samples with x = (a) 0.0, (b) 0.1, (c) 0.2, (d) 0.3, (e) 0.6, (f) 0.8, (g) 0.9, and (h) 1.0. Background has been removed for clearer representation.

Table S2: Phase quantity and lattice parameters calculated through Rietveld refinement for samples with multiphase and hexagonal (*$P6_3mc$*) crystal structure:

**Sample**	***x* = 0.8**	***x* = 0.9**	***x* = 1.0**
Nominal composition	$Ca_{1.05}AgSb_{0.2}Bi_{0.8}$	$Ca_{1.05}AgSb_{0.1}Bi_{0.9}$	$Ca_{1.05}AgBi$
Rwp (%)	2.28	4.26	4.94
Phases (wt. %)	*Pnma*: 30.47(18),<br>$P6_3mc$: 59.4(2),<br>Ag: 5.40(7),<br>Ca_2Bi: 4.77(17)	$P6_3mc$: 94.89(11),<br>Ag: 2.56(11),<br>CaAg: 2.558(7)	$P6_3mc$: 84.8(2),<br>Ag: 5.71(13),<br>CaAg: 9.5(2)
Pnma Lattice parameters (Å)	a: 7.8688(18)<br>b: 4.6508(13)<br>c: 8.5305(12)	-	-
$P6_3mc$ Lattice parameters (Å)	a: 4.7928(2)<br>c: 7.8584(7)	a: 4.8006(3)<br>c: 7.8613(8)	a: 4.811491<br>c: 7.868309

SI 3: Elastic constants vs composition and temperature:

The evolution of shear and bulk modulus is shown in Figure S3 as a function of composition. Shear modulus follows the same trend as Young's modulus, showing ~7% stiffening at the structural transition. Bulk Modulus for the hexagonal samples is significantly lower than that in the orthorhombic structure.

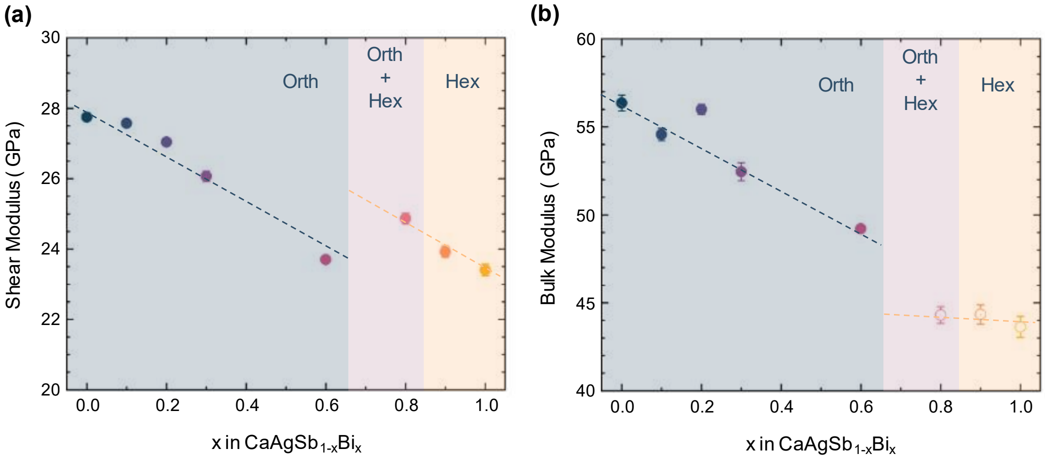

Figure S3: Evolution of the room temperature (a) shear and (b) bulk elastic moduli as a function of composition, *x*. The bulk modulus decreases slightly in the hexagonal phase, which is the result of the Poisson ratio decreasing.

For samples x = 0.0, 0.1, 0.6, and 1.0, elastic constants were determined as a function of temperature. The results show monotonous softening as a function of temperature, as seen in Figure S4.

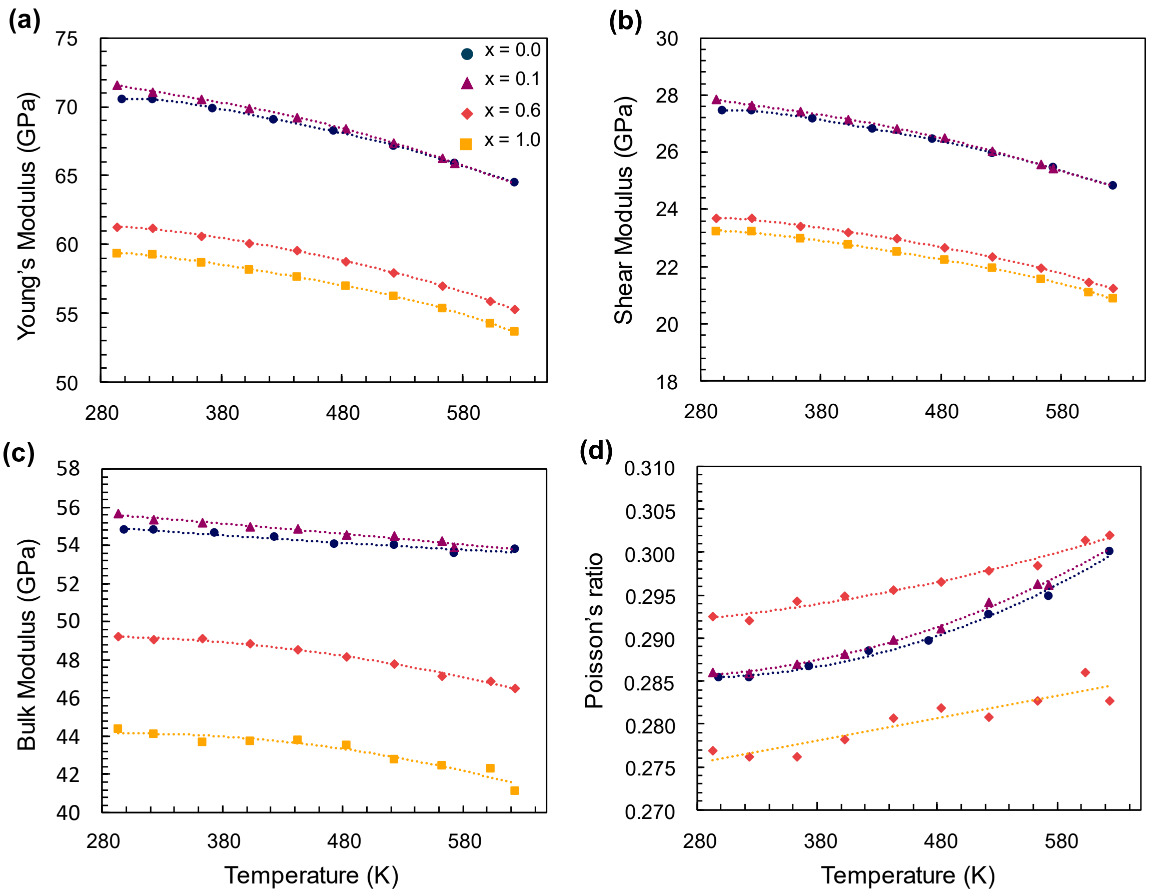

Figure S4: Temperature-dependent (a) Young's modulus, (b) shear modulus, (c) bulk modulus and (d) Poisson's ratio for x = 0.0, 0.1, 0.6 and 1.0. Only heating results are shown here.

SI 4: Debye temperature and glassy limit:

Table S3 shows the density, Rule-of-Mixture(ROM) relative densities, and volume per atom (V_a) calculated from lattice parameters for SPS'ed pellets. The longitudinal and transverse speeds of sound, v_L and v_T respectively, are calculated using RUS. The mean speed of sound (v_m) is calculated using the equation[1,2]:

$$\frac{1}{v_m^3} = \frac{1}{3}\left(\frac{2}{v_T^3} + \frac{1}{v_L^3}\right)$$

The Debye temperature is then calculated using the equation[2]:

$$\theta_D = \frac{h}{k_B}\left(\frac{3}{4\pi V_a}\right)^{1/3} v_m$$

The Cahill minimum lattice thermal conductivity, also called the glassy or amorphous limit, is calculated using the equation[3]:

$$\kappa_{glass} = \frac{1}{2}\left(\frac{\pi}{6}\right)^{1/3} k_B V^{-2/3}(2\nu_T + \nu_L)$$

The diffuson model by Agne *et al.* was used to calculate the minimum thermal conductivity κ_{diff} using the equation[4]:

$$\kappa_{diff} = \frac{0.76}{3} k_B V^{-2/3}(2\nu_T + \nu_L)$$

Table S3: Select properties across the solid solution $CaAgSb_{1-x}Bi_x$.

x	Density (g/cm^3)	ROM relative density (%)	v_L (m/s)	v_T (m/s)	v_m (m/s)	Volume per atom ($Å^3$)	Debye Temperature (K)	κ_{glass} (W/mK)	κ_{diff} (W/mK)
0.0	5.93	98.28	3969	2164	2413.6	25.00	245.7	0.54	0.34
0.1	6.09	98.58	3871	2127	2371.0	25.01	241.4	0.53	0.33
0.2	6.22	98.27	3846	2084	2325.5	25.22	236.1	0.52	0.33
0.3	6.39	98.50	3695	2020	2252.6	25.37	228.2	0.5	0.31
0.6	6.71	96.61	3471	1880	2097.9	26.13	210.5	0.46	0.29
0.8	6.89	95.14	3352	1899	2111.7	25.86	212.6	0.45	0.29
0.9	7.01	94.73	3298	1848	2056.8	26.13	206.4	0.44	0.28
1.0	7.13	94.47	3239	1812	2017.0	26.29	201.9	0.43	0.27

SI 5: Comparison of thermoelectric transport properties of CaAgSb

Prior to this work, the thermoelectric properties of undoped CaAgSb were reported in reference 30, where the samples were synthesized through a Pb-flux. Here, we compare the thermoelectric properties of CaAgSb in this work to the flux grown CaAgSb results in reference 30.

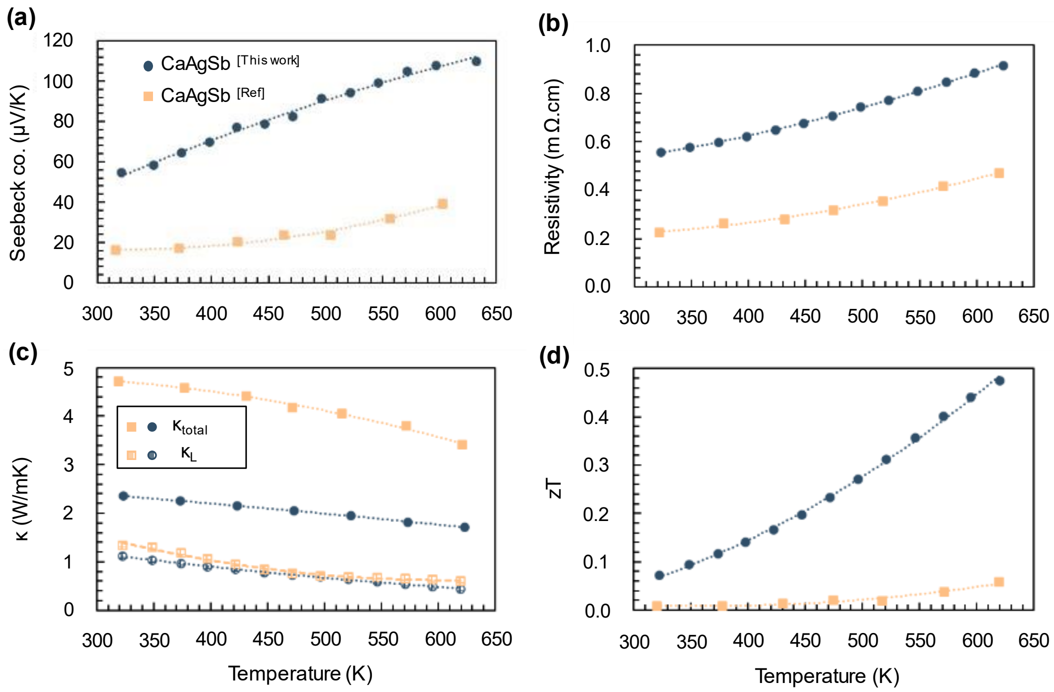

Figure S5: TE properties of CaAgSb found in this work when compared to that reported in ref 30. (a) Seebeck coefficient and (b) resistivity are seen to increase, suggesting lower of carrier concentration in the present work. This caused a decrease in (c) thermal conductivity and an overall increase in (d) *zT* by almost an order of magnitude. Lattice thermal conductivity was calculated using the Wiedemann-Franz Law where Lorenz number was determined using the empirical equation based on the Seebeck coefficient, L_{SPB}.

SI 6: Reversibility of transport properties:

To ensure reversibility of transport properties, measurements were carried out on each sample during both heating and cooling. The results are shown in Figure S6. Minimal hysteresis is observed in all measurements.

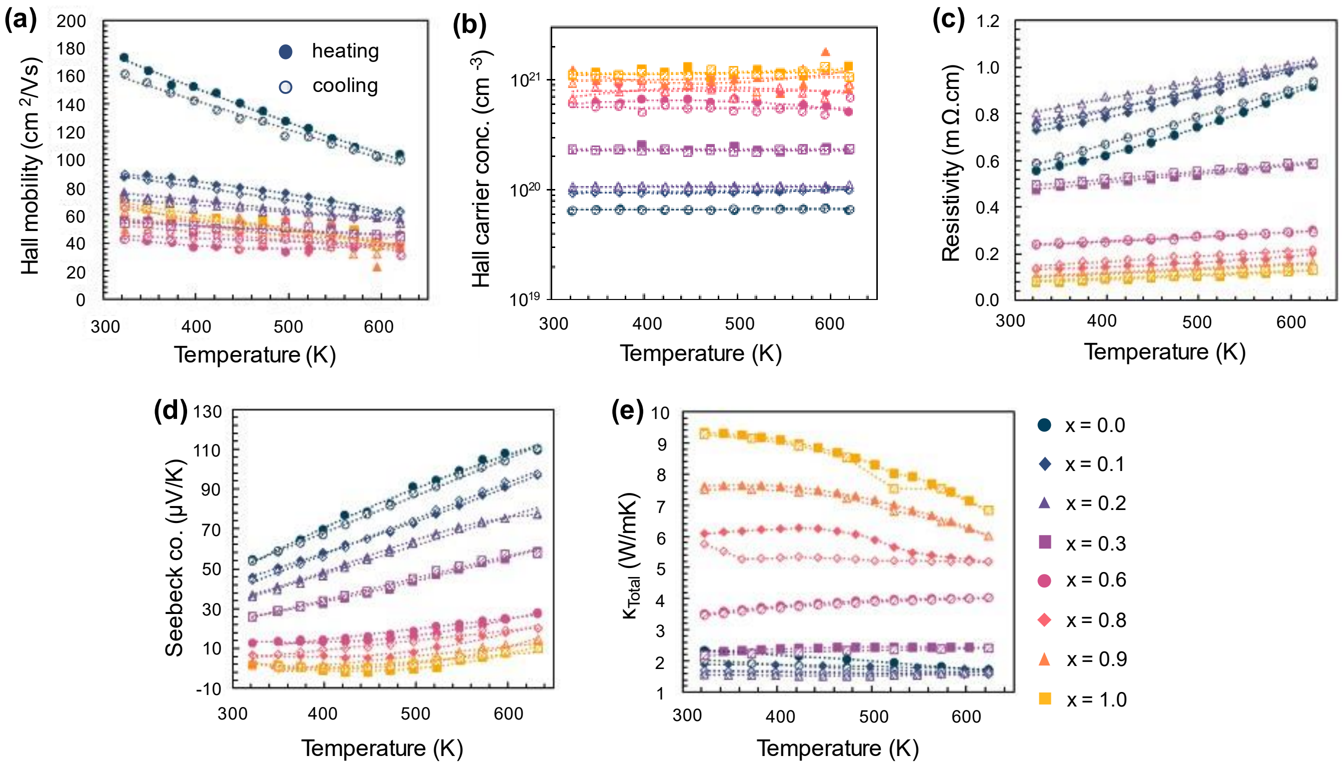

Figure S6: (a) Hall Mobility, (b) carrier concentration, (c) resistivity, (d) Seebeck coefficient, and (e) total thermal conductivity as a function of temperature. Solid symbols represent heating data and hollow, patterned symbols represent cooling data.

SI 7: Spin-orbit coupling effects on band structure:

For heavy elements such as Sb and Bi, spin-orbit coupling (SOC) can play a role in the band structure and subsequent properties. To probe the effects, DFT calculations were carried out to include spin-orbit interactions. Figure S7 shows a comparison between PBE and PBE+SOC band structures. There is little-to-no difference between the two band structures in CaAgSb. However, in the case of CaAgBi, SOC reveals inverted band crossings in the Γ – M and Γ – K directions. The results are consistent with what was seen by Sasmal *et al*[5].

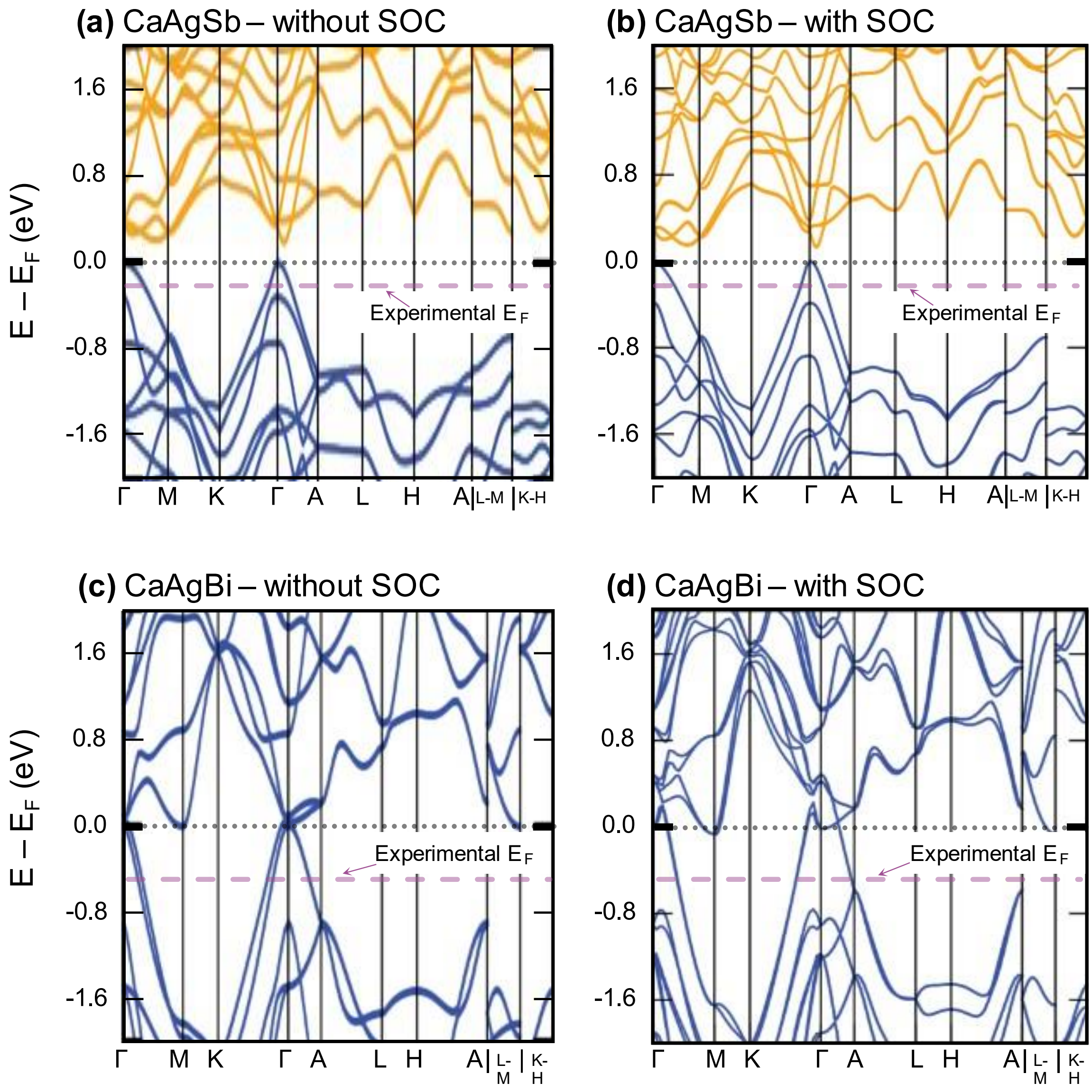

Figure S7: Band structures for CaAgSb show no difference (a) without SOC or (b) with SOC. For CaAgBi, there is a large difference between (c) without SOC and (d) with SOC band structures, as band inversion is seen when SOC is included in the calculations.

SI 8: Electronic and lattice thermal conductivity estimated using the SPB model:

Traditionally, electronic thermal conductivity is calculated using the Weidemann-Franz law, where the Lorenz number has certain assumptions built into it. Using the SPB-model and assuming that acoustic deformation potential scattering is dominant, Lorenz number can be extrapolated using the equation $L_{SPB} = 1.5 + exp^{\left(-|S|/_{116}\right)}$, which has been used extensively in literature. Figure S8 shows the electronic thermal conductivities (κ_E) for all values of x in $CaAgSb_{1-x}Bi_x$, followed by the lattice thermal conductivity (κ_L), calculated by subtracting electronic contributions from total thermal conductivity. Eminently, the lattice thermal conductivity is found to be negative for Bi-rich samples through this approach.

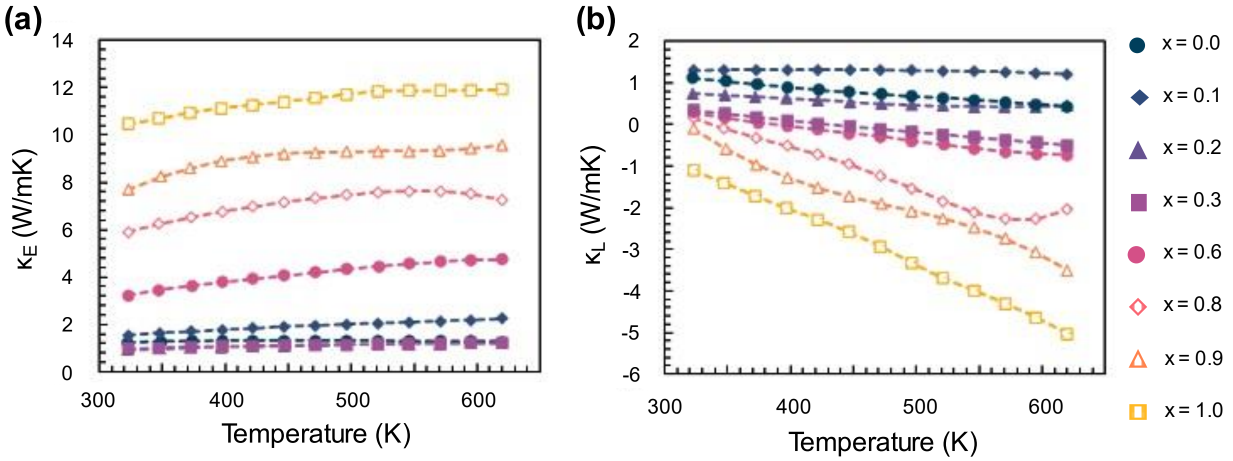

Figure S8: Temperature-dependent trends for (a) electronic (κ_E) and (b) lattice (κ_L) contributions to thermal conductivity. The Lorenz number was calculated from the single-parabolic band model using the equation $L_{SPB} = 1.5 + exp^{\left(-|S|/_{116}\right)}$. The results are shown based on heating data only.

SI 9: Landauer formalism to get Lorenz number:

In the diffusive limit Landauer formalism, the transport distribution function, G(E), is given as:

$$G(E) = \langle \nu(E) \rangle^2 . \tau(E) . g(E)$$

where *ν(E)*, *τ(E)*, and *g(E)* denote the energy-dependent carrier velocity, relaxation time, and density of states (DOS) respectively. For this work, we assume that the carrier relaxation time *τ(E)* scales inversely to the magnitude of the DOS, which leads to the equation: $\tau(E) = {}^{\tau_0}/_{g(E)}$, where τ_0 is the relaxation time scaling factor. This turns the *G(E)* equation to:

$$G(E) = \langle \nu(E) \rangle^2 . \tau_0$$

At a given temperature, the integral for Seebeck coefficient is solved for experimental Seebeck coefficient to determine the Fermi energy level E_F. The Seebeck integral can be written as:

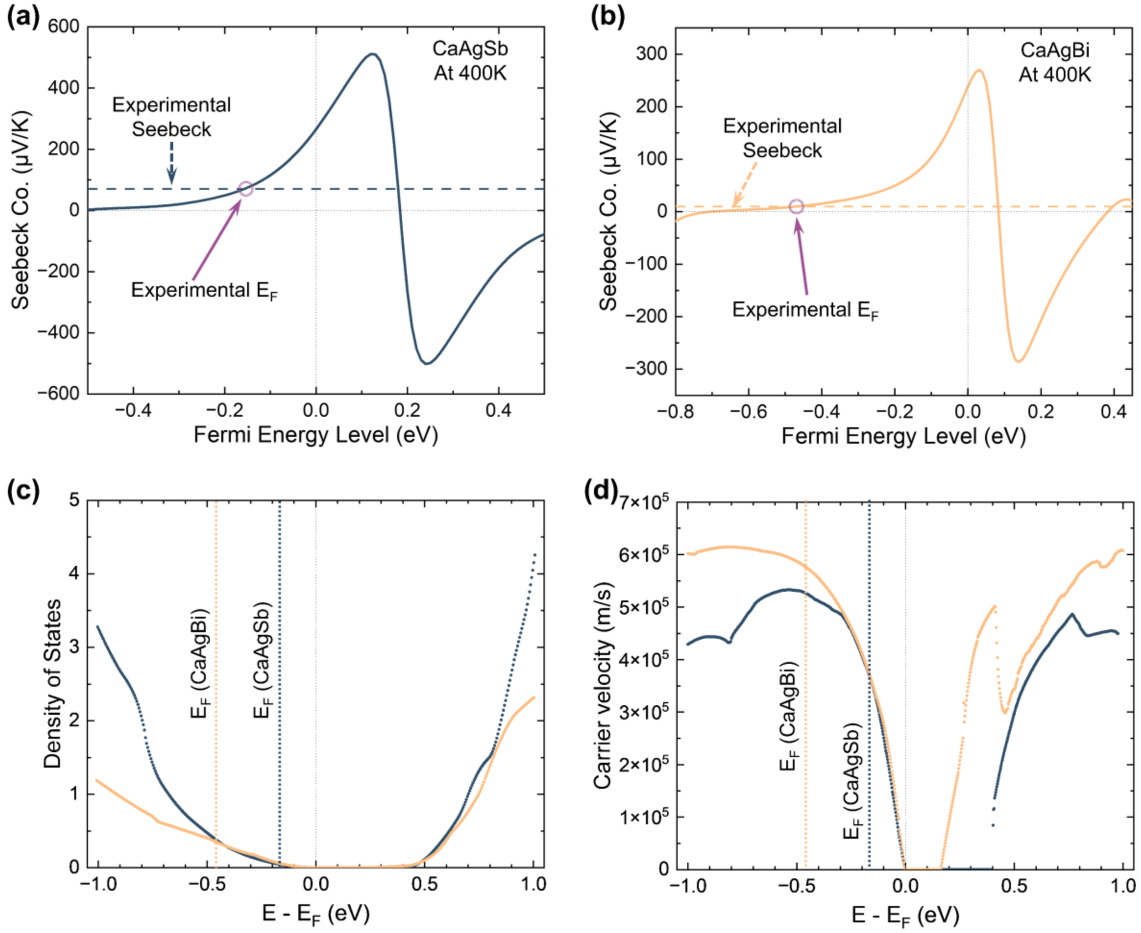

Figure S9: Seebeck coefficient vs. energy integrand used to find the position of E_F at 400 K for (a) CaAgSb and (b) CaAgBi using experimental values of Seebeck coefficient. (c) Density of states and (d) carrier velocity calculated from DFT shows the position of the determined E_F at 400 K. For CaAgSb, E_F lies at 0.16 eV below the valence band maximum, while that for CaAgBi lies at 0.46 eV.

$$S = \frac{\int_{-\infty}^{\infty} G(E) \frac{-\partial f}{\partial E} \frac{(E - E_F)}{T} dE}{\int_{-\infty}^{\infty} G(E) \frac{-\partial f}{\partial E} dE}$$

Where $\frac{\partial f}{\partial E}$ is the derivative of Fermi-Dirac distribution. Note that the τ_0 term cancels in consideration of the Seebeck coefficient within these approximations. SI Figure 7 shows a representation of how the integral was used to determine E_F at 400K for the two compositions. This places the Fermi energy level for CaAgSb close the valence band edge, while for CaAgBi, the E_F is predicted to be deep within the band, as shown in Figure S9.

Subsequently, the integral for electrical conductivity is used to determine $\tau_0(T)$ using the experimental temperature-dependent conductivity, which can be written as:

$$\sigma = q^2 \int_{-\infty}^{\infty} G(E) \frac{-\partial f}{\partial E} dE$$

Consequently, $\tau_0(T)$ gets its temperature dependance from experimental values of conductivity, as shown in Figure S10(a). $\tau_0(T)$ and E_F are then used to calculate $\tau(E)$ at different temperatures. At 400K, the energy dependence of τ is shown in Figure S10(b).

Once all the variables are calculated, the short circuit electronic thermal conductivity κ_0 integral is solved to produce κ_E as a function of temperature. The equations are given below. The results are shown in Figure S10(c).

$$\kappa_E = \kappa_0 - S^2 \sigma T$$

$$\kappa_0 = \int_{-\infty}^{\infty} G(E) \frac{-\partial f}{\partial E} \frac{(E - E_F)^2}{T} dE$$

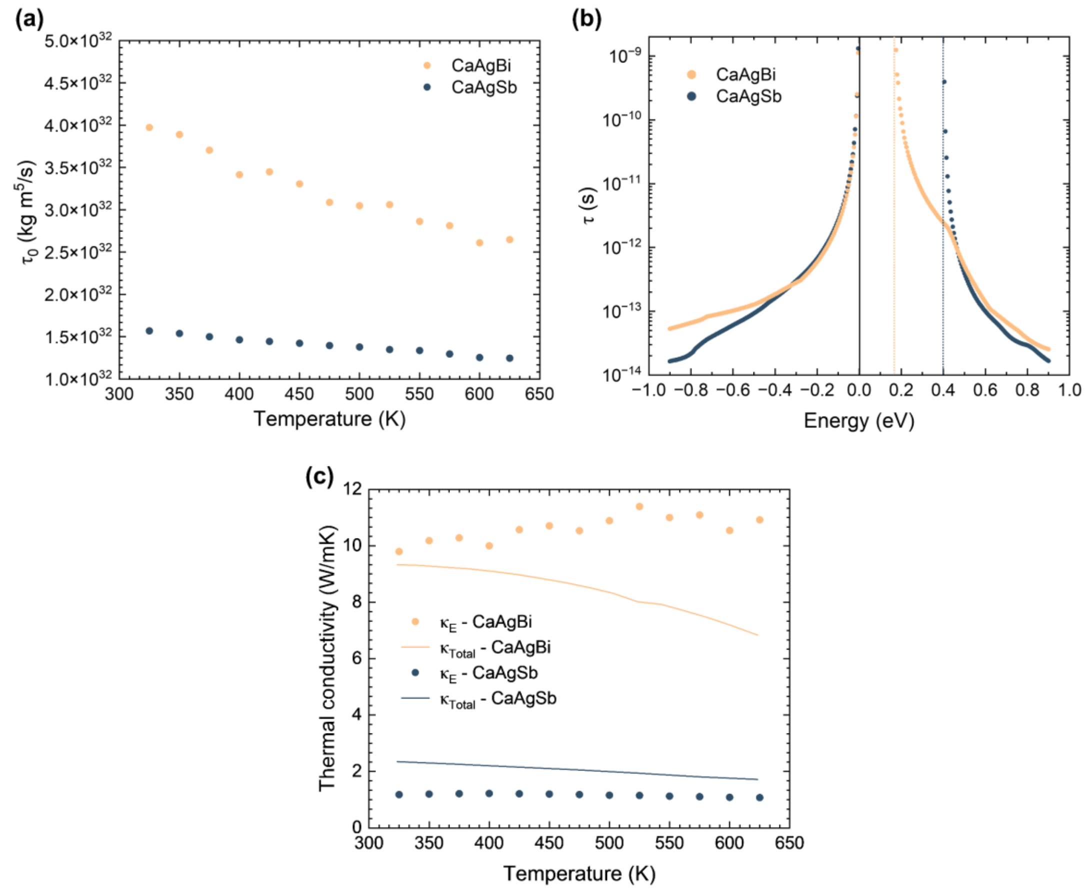

Figure S10: (a) $\tau_0(T)$ for the two end members and (b) Isothermal relaxation time as a function of energy at 400 K. (c) Electronic thermal conductivity calculated for both end members are shown along with experimental total thermal conductivity as a function of temperature.

SI 10: Isothermal Lorenz number:

The Weidemann-Franz law can also be written as $\kappa_{Total} = \kappa_L + \sigma \; x \; T \; x \; L$, which represents the equation of a straight line. The isothermal Lorenz number model assumes that at a given temperature, the proportionality constant remains constant for a given series of materials. As such, the proportionality constant and therefore the Lorenz number can be calculated from linear fit of total thermal conductivity vs electrical conductivity plot. Linear fits of the data at different temperatures are shown in Figure S10, where Lorenz number ($L_{Isothermal}$) is calculated from the slope. Linear extrapolation of L was done as a function of temperature and the values were put in the Weidemann-Franz law equation ($\kappa_E = \sigma . T . L_{Isothermal}$,) to calculate electronic contributions to thermal conductivity. Subtracting κ_E from κ_{Total}, κ_L was calculated, as shown in SI Figure 10.

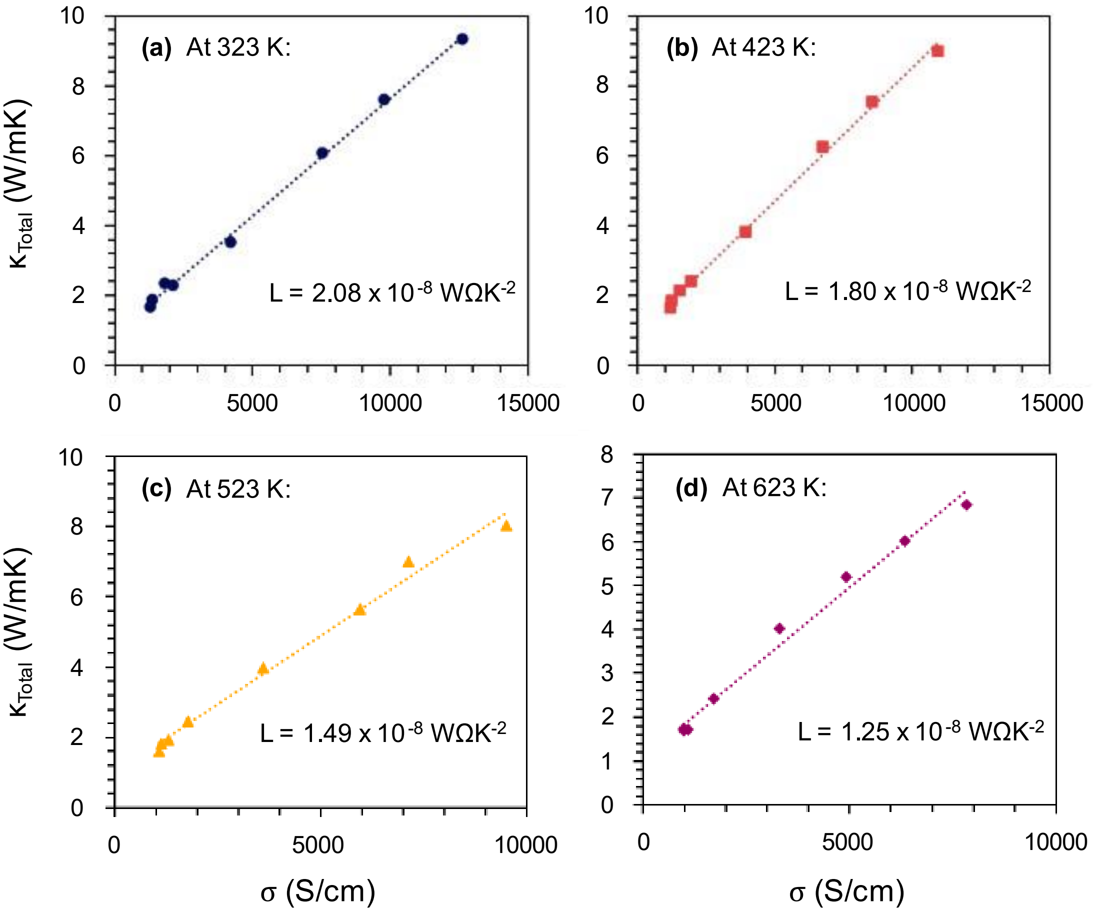

Figure S11: Experimental total thermal conductivity vs. electrical conductivity, used to determine values of the Isothermal Lorenz number at (a) 323 K, (b) 423 K, (c) 523 K, and (d) 623 K. According to the Wiedemann-Franz Law, the graphs are fit to the equation $\kappa_{Total} = \kappa_L + \sigma \; x \; T \; x \; L$ and the Lorenz number, $L_{Isothermal}$ is calculated from the slope at each temperature.

$L_{Isothermal}$ was used to calculate electronic thermal conductivity and lattice thermal conductivity using the Weidemann-Franz law. Positive values of κ_L were found for all samples over the entire temperature range, as shown in Figure S11.

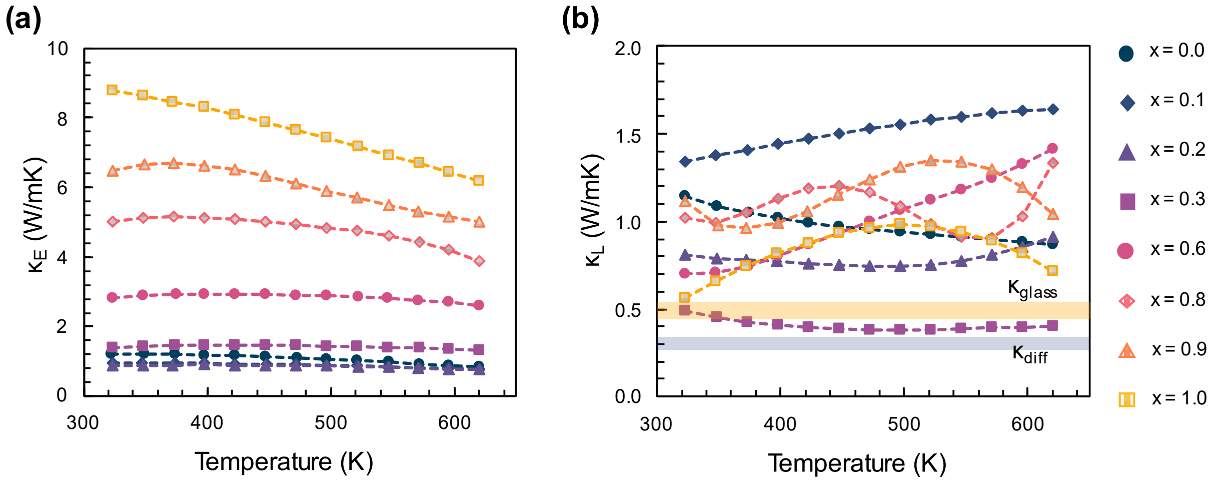

Figure S12: (a) Electronic contributions and (b) lattice contributions to thermal conductivity calculated using the isothermal Lorenz number model. Results were taken based on heating data only.

SI 11: Electronic transport along different directions:

In this study, the thermal transport has been measured parallel to the pressing direction while resistivity is measured perpendicular to the pressing direction. To ensure that the properties are consistent in all directions, electronic transport properties were measured along both directions for CaAgBi-end member. A tall sample was prepared and cut along the two directions. Figure S13 shows that hall mobility and carrier concentration are similar along the two directions, despite some scatter. The resistivity varies between parallel and perpendicular direction by 10 to 20 %, which translates to a similar difference in electronic contribution to thermal conductivity. When L_{SPB} was used to calculate electronic and lattice contributions to thermal conductivity, despite the difference in directional electrical conductivity, lattice thermal conductivity was found to be negative throughout the entire temperature range.

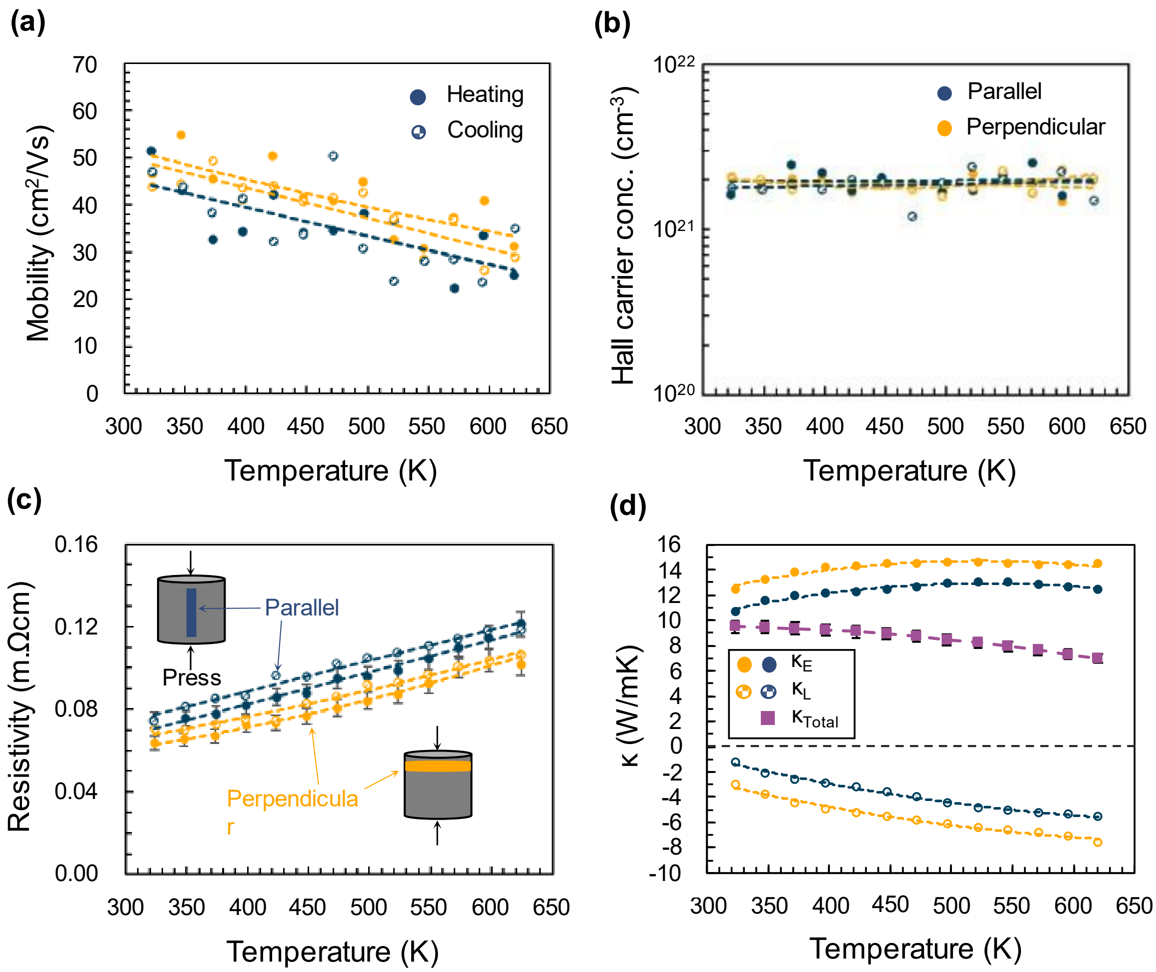

Figure S13: To assess anisotropy effects, van der Pauw measurements were conducted in both parallel and perpendicular directions to the pressing axes for CaAgBi. Both (a) hall mobility and (b) carrier concentrations were found to be almost identical within the scatter. (c) Resistivity results show deviations of 10 to 20% between the two directions. (d) The electronic and lattice contributions to thermal conductivity were calculated using L_{SPB} and the Weidemann-Franz Law, and the 10-20% difference is seen in both results across the two directions. Despite the difference, κ_L remains negative across the entire temperature range for CaAgBi. Note that κ_{Total} is calculated from thermal diffusivity measured parallel to the pressing direction.

References

(1) Zevalkink, A.; Smiadak, D. M.; Blackburn, J. L.; Ferguson, A. J.; Chabinyc, M. L.; Delaire, O.; Wang, J.; Kovnir, K.; Martin, J.; Schelhas, L. T.; Sparks, T. D.; Kang, S. D.; Dylla, M. T.; Snyder, G. J.; Ortiz, B. R.; Toberer, E. S. A Practical Field Guide to Thermoelectrics: Fundamentals, Synthesis, and Characterization. *Appl. Phys. Rev.* **2018**, *5* (2), 21303. https://doi.org/10.1063/1.5021094.

(2) Suzuki, Y.; Levine, J. B.; Migliori, A.; Garrett, J. D.; Kaner, R. B.; Fanelli, V. R.; Betts, J. B. Rhenium Diboride's Monocrystal Elastic Constants, 308 to 5 K. *J. Acoust. Soc. Am.* **2010**, *127* (5), 2797–2801. https://doi.org/10.1121/1.3372629.

(3) Cahill, D. G.; Watson, S. K.; Pohl, R. O. Lower Limit to the Thermal Conductivity of Disordered Crystals. *Phys. Rev. B* **1992**, *46* (10), 6131–6140. https://doi.org/10.1103/PhysRevB.46.6131.

(4) Agne, M. T.; Hanus, R.; Snyder, G. J. Minimum Thermal Conductivity in the Context of: Diffuson - Mediated Thermal Transport. *Energy Environ. Sci.* **2018**, *11* (3), 609–616. https://doi.org/10.1039/c7ee03256k.

(5) Sasmal, S.; Mondal, R.; Kulkarni, R.; Thamizhavel, A.; Singh, B. Magnetotransport Properties of Noncentrosymmetric CaAgBi Single Crystal. *J. Phys. Condens. Matter* **2020**, *32* (33), 335701. https://doi.org/10.1088/1361-648X/ab8520.
